# Supplementary material for: The short-term impact of 3 smoked cannabis preparations versus placebo on PTSD symptoms: A randomized cross-over clinical trial
Source: PLoS One. 2021 Mar 17;16(3):e0246990. doi: 10.1371/journal.pone.0246990 (PMC7968689; doi:10.1371/journal.pone.0246990)
Supplement: S13 File — (PDF) [file pone.0246990.s015.pdf]

PROTOCOL MJP-1

IND #110513

Original: November 5, 2010
Amendment 1: March 9, 2011
Amendment 2 Version 1: September 14, 2012
Amendment 2 Version 2: October 11, 2012
Amendment 2 Version 3: December 19, 2014
Amendment 3 Version 1: April 7, 2015
Amendment 3 Version 2: June 8, 2015
Amendment 3 Version 3: August 3, 2015
Amendment 3 Version 4: September 1, 2015
Amendment 4 Version 1: October 13, 2015
Amendment 5 Version 1: June 28, 2016
Amendment 6 Version 1: March 16, 2017
Amendment 7 Version 1: July 10, 2017
Amendment 8 Version 1: November 29, 2017
Amendment 8 Version 2: December 4, 2017

Placebo-Controlled, Triple-Blind, Randomized Crossover Pilot Study of the Safety and Efficacy of Four Different Potencies of Smoked Marijuana in 76 Veterans with Posttraumatic Stress Disorder (PTSD)

**SPONSOR**	Multidisciplinary Association for Psychedelic Studies (MAPS)<br>1115 Mission Street<br>Santa Cruz, CA 95060<br>Phone: 831-429-6362
**SPONSOR DESIGNEE**	Amy Emerson<br>MAPS Public Benefit Corporation (MPBC)<br>Phone: 510-393-7224

**COORDINATING INVESTIGATOR**	Marcel O. Bonn-Miller Ph.D.<br>Adjunct Assistant Professor of Psychology<br>Department of Psychiatry<br>University of Pennsylvania<br>3440 Market Street, Suite 370<br>Philadelphia, PA 19104<br>Phone: 650-493-5000 ext. 27908
**SITE INVESTIGATORS**	Site 01: Arizona<br>Suzanne Sisley, M.D.<br>1225 West Deer Valley Road<br>Phoenix, AZ 85027<br>Phone: 480-326-6023
**MEDICAL MONITORS**	Julie Holland<br>200 East 33rd Street, Suite 16H<br>New York, NY 10016<br>Phone: 212-358-5808<br><br>Michael C. Mithoefer, M.D.<br>Assistant Clinical Professor of Psychiatry<br>Medical University of South Carolina<br>208 Scott Street<br>Mt. Pleasant, SC<br>Phone: 843-849-6899

Table of Contents

1.0 List of Abbreviations 5
2.0 Introduction 7
2.1 Protocol Purpose 7
2.2 Supporting Information 8
2.2.1 Condition 8
2.2.2 Marijuana as a Treatment for PTSD Symptoms 9
2.3 Rationale for Concentration Selection 11
3.0 Protocol Objectives 12
3.1 Primary Objective 12
3.2 Secondary Objectives 12
3.3 Safety Objectives 13
3.4 Process Objectives 14
4.0 Protocol Design 14
4.1 Planned Duration of Study 15
4.2 Randomization and Participant Numbering 15
4.3 Recruitment and Participant Population 16
4.3.1 Participant Inclusion Criteria 17
4.3.2 Participant Exclusion Criteria 18
5.0 Methods 18
5.1 Assessments and Measures 18
5.1.1 Outcome Measures 18
5.1.2 Safety Measures 20
5.1.3 Process Measures 22
5.2 Study Procedures and Visit Descriptions 27
5.2.1 Pre-screening 27
5.2.2 Screen 1 27
5.2.3 Screen 2 29
5.2.4 Enrollment 29
5.2.5 Introductory Sessions in Stage 1 (Visit 1 and Visit 2) and Stage 2 (Visit 8 and Visit 9) 30
5.2.6 Daily Contact after Introductory Sessions 32
5.2.7 Self Administration During Stage 1 and Stage 2 32
5.2.8 Weekly Evaluations During Stage 1 (Visit 3 and Visit 4) and Stage 2 (Visit 10 and Visit 11) 33
5.2.9 Primary and Secondary Endpoint Evaluations in Stage 1 (Visit 5) and Stage 2 (Visit 12) 34
5.2.10 Two Weeks of Abstinence from Marijuana Self-Administration during Cessation 1 (Visit 6) and Cessation 2 (Visit 13) 34
5.2.11 Re-Baseline Evaluation at End of Cessation 1 (Visit 7) and Cessation 2 Final Outcome (Visit 14) 35
5.2.12 Stage 3 36
5.3 Removal of Enrolled Participants from the Study 37
5.4 Premature Discontinuation of the Study 37
6.0 Investigational Product 37
6.1 Substance Description and Activity Related to Proposed Action 37

6.2 Substance Concentrations, Packaging and Labeling 38
6.2.1 Doses 38
6.2.2 Packaging 39
6.2.3 Labeling 40
6.3 Substance Accountability 40
6.4 Substance Storage and Handling 40
6.5 Substance Stability 41
7.0 Risks of Participation 41
7.1 Risks of Screening, Study Procedures, Assessments and Measures 41
7.2 Risks of Self-administering Marijuana 42
7.3 Risk Mitigation 46
7.4 Medical Emergencies 49
8.0 Adverse Events 50
8.1 Spontaneously Reported Reactions 51
8.2 Serious Adverse Events 51
8.3 Adverse Event Collection 52
8.4 Adverse Event Reporting 53
9.0 Collection of Concomitant Medications and Therapies 54
10.0 Laboratory Assessments 54
10.1 Clinical Laboratory Assessments 54
10.2 Blood Cannabinoid Analysis 56
10.3 Research Lab Biomarker Analysis 56
11.0 Study Monitoring, Auditing and Documentation 57
12.0 Data Analysis 57
12.1 Statistical Power 58
13.0 Informed Consent 59
13.1 Confidentiality 60
13.2 Costs to Participants and Compensation 61
13.3 Treatment and Compensation for Study Related Injury 61
14.0 Record Retention 62
15.0 Publication Policy 62
16.0 References 63

1.0 List of Abbreviations

ACT	Actigraphy
AE(s)	Adverse Event(s)
ALT/SGPT	Alanine aminotransferase
AMI	Acute Myocardial Infarction
AST/SGOT	Aspartate aminotransferase
BAI	Beck Anxiety Inventory
C	Celsius
CAP	College of American Pathologists
CAPS	Clinician Administered PTSD Scale for DSM-5
CBD	Cannabidiol
CB1	Cannabinoid Receptor 1
CB2	Cannabinoid Receptor 2
CCK	Cholecystokinin
CI	Clinical Investigator (e.g. lead investigator, co-investigators)
CLIA	Clinical Laboratory Improvement Amendment
CPT	Cognitive Processing Therapy
CRA	Clinical Research Associate
CRF(s)	Case Report Form(s)
CRP	C-Reactive Protein
C-SSRS	Columbia Suicide Severity Rating Scale
CUDIT-R	Cannabis Use Disorders Identification Test-Revised
DBP	Diastolic Blood Pressure
DDIS	Dissociative Disorders Interview Schedule for DSM-5
DEA	Drug Enforcement Administration
DEQ	Drug Experiences Questionnaire
DMF	Drug Master File
DSM-5	Diagnostic and Statistical Manual of Mental Disorders – 5
ECG/EKG	Electrocardiogram
EDC	Electronic Data Capture
EMDR	Eye Movement Desensitization and Reprocessing
ePRO	Electronic Patient Reported Outcomes
EtG	Ethyl Glucuronide
F	Fahrenheit
FDA	Food and Drug Administration
GABA	Gamma-aminobutyric acid
GCP	Good Clinical Practice
HCL	Hydrochloric acid
HIPAA	Health Insurance Portability and Accountability Act
HIV	Human Immunodeficiency Virus
5-HT	5-Hydroxytryptamine
ICF	Informed Consent Form
ICH	International Conference on Harmonization
IDAS	Inventory of Depression and Anxiety Symptoms
IL-1β	Interleukin-1beta
IL-6	Interleukin-6

IND	Investigational New Drug
IPF	Inventory of Psychosocial Functioning
IR	Independent Rater
IRB	Institutional Review Board
IRS	Internal Revenue Service
ISF	Investigator Site File
ISI	Insomnia Severity Index
MAOI	Monoamine Oxidase Inhibitor
MAPS	Multidisciplinary Association for Psychedelic Studies
MDMA	3,4-methylenedioxymethamphetamine
MI	Myocardial infarction
MWC	Marijuana Withdrawal Checklist
NCT	National Clinical Trial Registry
NIDA	National Institute on Drug Abuse
OEF	Operation Enduring Freedom
OIF	Operation Iraqi Freedom
PCL-5	Posttraumatic Symptom Checklist based on DSM-5
PE	Prolonged Exposure
PTSD	Posttraumatic Stress Disorder
RCT	Randomized Controlled Trial
SAE(s)	Serious Adverse Event(s)
SBP	Systolic Blood Pressure
SCID-RV	Structured Clinical Interview for DSM-5 Diagnoses Research Version
SE	Sleep Efficiency
SNRI	Serotonin Norepinephrine Reuptake Inhibitor
SOL	Sleep Onset Latency
SOP(s)	Standard Operating Procedure(s)
SSRI	Selective Serotonin Reuptake Inhibitor
Stage 1	The initial, fully randomized, blinded study arm
Stage 2	The second, randomized, blinded study arm
Stage 3	The optional third study arm
THC	Δ-9-tetrahydrocannabinol
TLFB	Time-Line Follow-Back
TST	Total Sleep time
US	United States of America
VA	Veterans Administration
VAMM	Veterans Alliance for Medical Marijuana
WASO	Wake After Sleep Onsent
WDS	Withdrawal Discomfort Score

2.0 Introduction

This study is sponsored by the Multidisciplinary Association for Psychedelic Studies (MAPS), a U.S.-based, IRS-approved, 501(c)(3) non-profit research and educational organization. MAPS sponsors clinical research evaluating the potential of psychedelics and marijuana to become FDA-approved prescription medicines. MAPS is currently sponsoring a series of Phase 1/2/3 studies in the US and internationally for posttraumatic stress disorder (PTSD). The ongoing clinical development program includes multiple active protocols investigating the therapeutic potential of 3,4-methylenedioxymethamphetamine (MDMA)-assisted psychotherapy in treating people with PTSD. Results from two MAPS-supported studies of MDMA-assisted psychotherapy for PTSD have recently been published and include indications that the treatment can create durable improvements in PTSD symptom severity [1-3]. This study will continue MAPS' investigation into the treatment of PTSD with the first randomized controlled trial (RCT) to test the therapeutic potential of smoked marijuana and its components as a treatment for PTSD. This study is essential for understanding potential risks and therapeutic benefits of marijuana for PTSD patients.

MAPS is seeking to conduct the marijuana/PTSD pilot study described in this protocol in response to many reports of marijuana attenuating PTSD symptom expression among individuals with PTSD, including veterans PTSD [4]. As of December 2014, 23 states and the District of Columbia have medical marijuana legislation, and seven of these list PTSD as a qualifying condition for medical marijuana, including Arizona[5]. As of August 18, 2010, 633 of a total of 2316 individuals enrolled in the New Mexico medical marijuana program (27%) list PTSD as the primary condition they seek to treat with medical marijuana [6], making it the most common reason for enrollment. Similar prevalence has been observed in other states and among other populations, including military veterans [7, 8]. At present, there are no published data from randomized, placebo-controlled studies of the risks and benefits of marijuana for participants with chronic PTSD from any cause.

In this groundbreaking randomized, triple-blind, placebo-controlled, crossover study, marijuana will be tested as a pharmacological agent to manage PTSD symptoms in 76 US military veterans. This will be the first controlled clinical trial testing the therapeutic potential of marijuana for treating PTSD, and is essential for understanding potential risks and therapeutic benefits of marijuana for PTSD patients. Results will provide physicians, patients, scientists and regulators with critical knowledge regarding whether marijuana benefits individuals with PTSD, whether adverse consequences occur, and the impact of the chemical composition of marijuana, specifically Δ-9-tetrahydrocannabinol (THC) and cannabidiol (CBD) content, on clinical outcomes.

2.1 Protocol Purpose

This pilot study will gather preliminary evidence of the safety and efficacy of four potencies of smoked marijuana to manage PTSD among veterans. By working with veterans, we address a national emergency and limit variability at the potential expense of generalizability. Further research will be needed to determine if these results will apply to

other groups of PTSD sufferers. Smoked marijuana will be tested in two stages of three weeks each (Stage 1 and Stage 2), with a two-week cessation after each stage, verified by urine cannabinoid analysis. The study will produce preliminary evidence to help elucidate the contribution of THC, CBD, or a combination of both constituents to potential attenuation of PTSD symptoms. This study is critically important for understanding the potential risks and benefits of marijuana as a treatment for PTSD and can inform the development of larger randomized controlled clinical trials. *Ad libitum* self-administration of smoked marijuana with a range of THC and CBD ratios will be used in this study to provide a naturalistic comparison that is generalizable to what many veterans are currently using to manage PTSD symptoms in states with legalized medical marijuana. Results will provide information regarding marijuana dosing, composition, side effects, and specific areas of benefit to clinicians and legislators considering marijuana as an acceptable treatment for PTSD. This study will also provide information on the predictive value and/or assessment of the impact of marijuana on selected biomarkers of inflammation, and effects of marijuana concentration upon these biomarkers as a measure of PTSD treatment response.

2.2 Supporting Information

2.2.1 Condition

PTSD is a serious, worldwide public health problem for which a wider array of effective treatments is needed. In the US, the lifetime prevalence of PTSD in the general population is between 6 and 10% [9, 10]. PTSD is common in other countries as well [11-15]. Incidence of PTSD in US Operation Iraqi Freedom (OIF) and Operation Enduring Freedom (OEF) veterans is as high as 18% [16], and it is estimated that the number of service members returning home with PTSD will be between 75,000 and 225,000 [17]. In 2004, the US Veterans Administration (VA) spent $4.3 billion on PTSD disability payments to approximately 215,000 veterans, most of them from the Vietnam War [18]. Due to OIF/OEF, the number of veterans disabled by PTSD, and the cost of providing disability payments, has increased substantially since 2004. In countries where there is endemic armed conflict, the incidence of PTSD in civilians is often far greater [19-21]. PTSD is typically a chronic illness [22, 23], associated with high rates of psychiatric and medical co-morbidity, disability, suffering and suicide [13, 22, 24, 25]. An array of psychotherapeutic options exists for treating PTSD that include cognitive behavioral psychotherapies, such as Prolonged Exposure (PE), Cognitive Reprocessing Therapy (CPT), and therapies such as Eye Movement Desensitization and Reprocessing (EMDR) [26, 27], as well as two Selective Serotonin Reuptake Inhibitors (SSRIs; sertraline and paroxetine) that are approved as PTSD treatments by the FDA. However, a significant percentage of PTSD patients fail to respond adequately to established PTSD psychotherapies [28, 29], including SSRIs [30], or respond in ways that are statistically significant but clinically inadequate. At least one study of paroxetine indicated that men with PTSD did not respond to this drug [31]. These findings suggest that there is still substantial need for innovative treatments for PTSD.

There is limited data about whether or not patients with military service-related PTSD are more difficult to treat than those with PTSD from other causes. A recent comprehensive review found that, most, but not all, RCTs with combat (mostly Vietnam War) veterans showed less treatment efficacy than RCTs with non-veterans whose PTSD was related to other traumatic experiences. Therefore some experts believe that combat veterans with PTSD are less responsive to standard or widely used treatments. More clinical trials with combat veterans would be important and welcome additions to this literature [26].

Clinical reports suggest that patients with PTSD may have a tendency to favor depressants, such as alcohol, benzodiazepines, and marijuana to “self-medicate” symptoms of hyperarousal as opposed to stimulants (such as cocaine), which may exacerbate symptoms [32]. However, the extent to which illicit substances are used for symptom relieve in veterans with PTSD may be under-appreciated in clinical trials associated with the VA system [33-35]. Substance use in this group may be under-reported due to concerns that it might jeopardize potential claims for disability compensation or result in arrest or prosecution. Thus there is potential benefit of conducting studies of PTSD and substance use outside of the VA system that include multimodal assessments and measures of both PTSD and substance use.

2.2.2 Marijuana as a Treatment for PTSD Symptoms

The principal active component in the complex mixture of cannabinoids present in the marijuana plant is THC, which acts primarily as an agonist at the CB1 cannabinoid receptor. This receptor is found at high concentrations in the brain, including the basal ganglia and cerebellar regions, and also in the hippocampus and hypothalamus. THC has been shown to inhibit the release of a wide spectrum of neurotransmitters including L-glutamate, GABA, norepinephrine, dopamine, serotonin (5-HT), and acetylcholine [36]. Preliminary findings from assessing current and recalled PTSD symptoms in 80 patients suggests that use of medical marijuana can reduce PTSD symptoms [37], and a case study of a young man with PTSD assessed before and after he began using cannabis supports the therapeutic potential of marijuana for PTSD [38]. An open-label study in ten people reported that sublingual THC reduced PTSD symptoms, reduced nightmares and improved sleep quality [39]. Given evidence highlighting marijuana as a possible means of improving sleep [4], with retrospective reports of marijuana use and associated psychopathology indicating reductions in PTSD symptoms after use [37], it is not surprising that population-based studies have found PTSD to be associated with increased marijuana use [40]. These reports warrant further systematic investigation into marijuana as a potential adjunctive treatment for PTSD.

The presence of CB1 receptors in the hippocampus, amygdala, prefrontal and anterior cingulate cortex suggests that endocannabinoids are involved in regulating anxiety, attention to and response to stressful situations, and may be involved in the extinction of conditioned fear [41-43]. This hypothesis is supported by the observation that availability of CB1 receptors is particularly elevated among individuals with PTSD, compared to trauma-exposed or healthy controls [44]. Further support of this theory came from studies using CB1 receptor antagonists or CB1 receptor knockout mice. Several CB1 receptor agonists increased time mice spent in open arms of the elevated plus maze, a sign of

reduced anxiety [45], while CB1 receptor antagonist SR141716 produced anxiogenic effects in the elevated plus-maze and the defensive withdrawal tests in adult rats [46, 47]. Furthermore, another selective CB1 receptor antagonist, AM251, increased anxiety-like behavior in wild-type mice but had no effect in the knockouts, in support of a CB1 receptor-mediated anxiolysis.

Cannabinoids also modulate GABA-ergic transmission and the release of cholecystokinin (CCK), a peptide that may contribute to both anxiolytic and anxiogenic effects of THC and endocannabinoids [48-51]. Furthermore, cannabinoids enhance the release of endogenous opioids, and these may be involved in the functional interplay between the endocannabinoid and the opioid system and the production of analgesic responses. Some researchers hypothesize that the relationship between these two systems plays a role in antidepressant-like effects and in various addiction-related processes [52-54]. Studies in rodents suggest that cannabinoids and their interaction with endogenous opioids might also modulate anxiety [53, 55, 56]. Rodent studies detected antidepressant-like responses to CB1 agonists, and CB1 agonists can increase the synaptic concentration of biogenic amines, much like antidepressants do. Thus, pharmacological modulation of the endocannabinoid system holds considerable promise in the treatment of both anxiety-related and mood disorders.

The results of a recent study implicated endocannabinoids and CB1 receptors in the extinction of aversive memories by demonstrating that CB1 knockout mice show impaired extinction in auditory fear-conditioning tests, and this could be mimicked in wild-type mice by treatment with SR141716 [57], a finding supported through use of receptor agonists and antagonists [58, 59]. The synthetic cannabinoid nabilone was effective in reducing treatment-resistant nightmares in people with PTSD [60]. These findings raise the possibility that pharmacological amplification of CB1 signaling, for example, may have therapeutic value in obsessive-compulsive disorder or PTSD.

Benzodiazepines and related GABA agonists are frequently used to treat anxiety and sleep disruption in PTSD. Other treatments, such as the centrally acting adrenergic alpha1 antagonist prazosin, have been investigated as a treatment for PTSD-related sleep disorders [61]. However, these compounds can produce physical dependence and are not always well tolerated. Investigating alternate avenues of treatment for PTSD symptoms may lead to helping a greater number of individuals control their symptoms.

In addition to THC, whole plant marijuana contains a number of other active constituents, most notably CBD. Research indicates that THC acts upon receptors in brain areas involved in memory and fear processing, and preclinical studies in rodents suggest cannabinoids reduce fear [42, 45]. Research in mice found that CBD was comparable to the antidepressant imipramine in tests of antidepressant-like effects [62]. Findings of reduced PTSD symptoms, by orally administered THC or comparable synthetic cannabinoids, including nightmares and sleep difficulties suggest a potential role for cannabinoids in the alleviation of PTSD symptoms [63, 64]. Further, CBD may oppose anxiogenic effects of THC in humans [65, 66], and a naturalistic study found smoking marijuana with higher CBD levels was associated with less memory impairment and lower anxiety during intoxication [67]. It appears that CBD attenuates amygdalar

activation in response to facial expressions of fear in healthy participants [68], a potentially beneficial effect for people with PTSD, who may exhibit enhanced amygdalar reactivity to fearful faces [69]. Anecdotal reports from Veterans Alliance for Medical Marijuana (VAMM) indicate that veterans prefer a balanced THC to CBD intake for management of PTSD symptoms. These studies suggest that it is worth investigating the effects of marijuana that varies in THC and CBD content on the ability to suppress symptoms of PTSD.

Another potential mechanism by which marijuana may confer benefit in the treatment of PTSD relates to reductions in inflammation. A longitudinal study in US Marines reported an association between higher pre-deployment levels of C-reactive protein (CRP) and post-deployment development of PTSD [70]. In other research, reduction in PTSD symptoms following treatment with SSRIs was associated with a reduction in interleukin-1Beta (IL-1β) [71], and women whose PTSD symptoms were in remission exhibited lower levels of CRP and interleukin-6 (IL-6) compared with those with current PTSD [72]. Further, a meta-analysis supported a link between IL-1β and IL-6 and exposure to trauma [73], with stress-related elevation in IL-6 potentially higher among those with PTSD [72, 74]. Because both THC and CBD have potent anti-inflammatory and immunomodulatory properties [75, 76], marijuana use may have therapeutic benefit in PTSD treatment simply via reductions in inflammation. Biomarkers of inflammation will be assessed to investigate whether the anti-inflammatory properties of THC/CBD mediates the treatment effect of marijuana on PTSD symptom expression.

2.3 Rationale for Concentration Selection

Four potencies of marijuana obtained through the National Institute on Drug Abuse (NIDA) drug supply program that vary in THC and cannabidiol CBD content will be used: High THC/ Low CBD (High THC), Low THC/High CBD (High CBD), High THC/High CBD (THC/CBD) or Low THC/Low CBD (placebo) marijuana. “High” is defined as marijuana containing a target of 7-15% concentration by weight of the respective cannabinoid and “Low” is defined as <2% concentration by weight; actual cannabinoid concentrations are subject to the capabilities of the NIDA Drug Supply Program to produce different strains of marijuana based on these targets and are provided in Section 6. In the Stage 2 crossover, two of three concentrations will be compared: High THC, High CBD, and THC/CBD. *Ad libitum* self-administration will be permitted up to a maximum of 1.8 grams of marijuana/day.

The doses of marijuana (excluding placebo) in this study were chosen because they contain a range of THC and CBD ratios and potencies generalizable to what many veterans are currently using to manage PTSD symptoms in non-clinical settings in states with legalized medical marijuana. Prior to the study start, laboratory testing of the study marijuana will be completed to verify the chemical composition of each concentration group.

The active (non-placebo) doses are expected to produce commonly reported subjective effects of marijuana. The physiological effects are expected to be tolerable based on previous observational studies in the literature. The placebo is expected to produce

minimal side effects, without the full range of psychological and subjective effects seen with the active doses.

Analysis of the differential efficacy of these distinct marijuana concentrations will help elucidate whether the ratio of THC to CBD is a significant factor in the attenuation of PTSD symptoms, as reported anecdotally and based on the differential pharmacology of THC and CBD. *Ad libitum* use will provide the most naturalistic comparison to real world examples of smoked marijuana.

3.0 Protocol Objectives

The objectives of this study are:

1. To evaluate whether smoking whole plant marijuana attenuates PTSD symptoms.
2. To compare the efficacy of varying ratios of THC and CBD to placebo using standard clinical measures.
3. To collect safety data.

3.1 Primary Objective

To compare independent effects of three active concentrations of smoked marijuana and placebo on PTSD symptom severity measured by changes in CAPS-5 total scores during 3 weeks of *ad libitum* self-administration during Stage 1 of the study protocol.

3.2 Secondary Objectives

The following secondary objectives will be used to support the results of the primary objective:

1. Assess independent effects of three active concentrations of marijuana and placebo on changes from Baseline (enrollment) to Primary Endpoint in self-reported symptoms of PTSD (PCL-5), anxiety and depression (IDAS), and psychosocial functioning (IPF).
2. Assess independent effects of three active concentrations of marijuana and placebo on changes during self-administration periods in Stage 1 and Stage 2 in self-reported symptoms of PTSD (PCL-5), anxiety and depression (IDAS), and psychosocial functioning (IPF), pooling by marijuana concentration.
3. To compare independent effects of three active concentrations of marijuana and placebo on changes in PTSD symptom severity (CAPS-5) during self-administration periods in Stage 1 and Stage 2, pooling by marijuana concentration.
4. Assess whether participants' preferences for marijuana concentrations in Stage 1 and Stage 2 are predictive of changes in PTSD symptom severity (CAPS-5).
5. Assess whether participants experience a clinically significant change in PTSD symptoms based on CAPS-5 and PCL-5 total scores during Cessation 1 and 2 compared with end of Stage 1 and end of Stage 2.
6. Explore correlation of treatment outcomes measured by CAPS-5 score with

objective changes in sleep via Actigraphy and self-reported changes in sleep quality using the ISI, aggregated for the study periods of Stage 1, Cessation 1, Stage 2, and Cessation 2.

7. Explore correlation of objective changes in sleep measured via Actigraphy with self-reported changes in sleep quality using the ISI, aggregated to overlapping weekly periods.
8. Explore whether amount of marijuana used during self-administration in Stage 1 and Stage 2 is predictive of treatment outcomes on PTSD symptom severity (CAPS-5), within each marijuana concentration.

3.3 Safety Objectives

The safety of participants will be measured throughout the study by assessing physiological and subjective drug effects, psychological distress, adverse events (AEs), and suicidality, repeatedly as described in the following steps. Summaries of safety data by study stage will be provided to the Medical Monitors for review during the study.

1. Explore correlations of subjective effects with the DEQ and cardiovascular effects with heart rate of each active dose of marijuana and placebo during introductory sessions.
2. Explore independent effects of each active dose of marijuana and placebo on blood pressure and body temperature during introductory sessions.
3. Assess independent effects of each active dose of marijuana and placebo on marijuana withdrawal symptoms during screening and Cessation 1 and 2.
4. Assess independent effects of each active dose of marijuana and placebo on problems associated with marijuana use during the study, including follow-up, with the CUDIT-R.
5. Evaluate the subjective effects of each active dose of marijuana and placebo in controlled laboratory settings using the DEQ.
6. Assess independent effects of each active dose of marijuana and placebo on suicidality with the C-SSRS on a weekly basis throughout the treatment period, and more frequently if needed.
7. Assess independent effects of each active dose of marijuana and placebo on whether and for how many days participants were incarcerated throughout the study.
8. Assess independent effects of each active dose of marijuana and placebo on whether and for how many days participants were hospitalized throughout the study.
9. Assess metabolites of alcohol use through urine testing at face-to-face visits.
10. Collect, review, and report Serious Adverse Events (SAEs) and AEs in accordance with FDA regulations and Section 8.0 of the protocol.

3.4 Process Objectives

The following objectives will include exploratory analyses intended to inform future studies:

1. Evaluate protocol compliance based on comparison of Daily Diary entries, including information on amount, time and frequency of marijuana use, and the weight of any unused marijuana across each study stage.
2. Evaluate protocol compliance using quantitative blood and/or urine cannabinoid levels.
3. Evaluate whether markers of inflammation CRP, IL-1β, and IL-6 levels in blood predict PTSD severity at baseline and treatment outcome as a function of marijuana concentration.
4. Assess the ability of site staff and participants to accurately guess condition assignment in Stage 1 and Stage 2.

4.0 Protocol Design

This Phase 2 randomized, placebo-controlled, triple-blind, crossover study will assess the safety and efficacy of four types of smoked marijuana to manage PTSD symptoms among 76 veterans in an outpatient setting. The study will consist of a baseline period and three distinct study stages, which are described in detail below.

An initial 2-week screening period will be conducted to determine study eligibility prior to randomization, to obtain baseline measures of PTSD and substance use, to conduct study training, and ensure participants are willing and able to attend scheduled appointments and complete the study procedures.

Following randomization, participants will complete Stage 1, a three-week period of *ad libitum* marijuana self-administration followed by a two-week period of marijuana abstinence (Cessation 1). This will be immediately followed by Stage 2, another three-week period of marijuana self-administration and two-week period of abstinence (Cessation 2). Stage 1 and Stage 2 will consist of identical procedures and measures, but the marijuana available to individual participants will vary.

During Stage 1, participants will be randomized to receive High THC, High CBD, THC/CBD, or Placebo marijuana to self-administer (up to 1.8 g/day) for the 3 week marijuana use period. As described in Section 4.2, randomization will be balanced across drug conditions (N=19 per marijuana type; total N=76). During Stage 2, the Placebo marijuana condition will be removed and all participants will be re-randomized to receive a different type of marijuana than they were assigned in Stage 1. Thus, Stage 2 will permit a within-subjects comparison of changes in CAPS-5 total score as a function of marijuana concentration, including differences in preferences for a specific marijuana concentration by self-report, and the removal of placebo as a possibility ensures that all study participants receive active marijuana at some point during the study.

Following completion of Stage 2, participants will be invited to participate in an optional Stage 3. During Stage 3, participants can choose to receive the amount equivalent to the unused and returned marijuana from Stage 1 or Stage 2 from a new supply. This design feature discourages diversion of unused marijuana and encourages participant use of marijuana in a naturalistic manner during Stages 1 and 2. Marijuana will be dispensed during Stage 3 based on participant use during Stages 1 and 2 and will not last more than two months. Marijuana in Stage 3 will be packaged in the greatest single daily amount used from the chosen stage for weekly dispensing until the amount of all unused product is depleted.

4.1 Planned Duration of Study

Study duration for each participant will be two and a half months to four and a half months from the time the participant is enrolled in the study. The study will start after marijuana is obtained from the NIDA Drug Supply Program, and composition may be verified by an appropriately qualified laboratory. Assuming that eight participants can be enrolled each month, the study should take approximately two years to complete.

4.2 Randomization and Participant Numbering

To achieve sufficient statistical power and account for dropouts, about 116 participants will be stratified by site and randomized in a 1:1:1:1 ratio across the four treatment groups (High THC, High CBD, THC/CBD, or Placebo) based on a sequential order of enrollment into the study. Participants will be assigned a randomization code that will correspond to a blinded condition assignment. Randomization and enrollment will halt when 76 participants complete Stage 1 (at least N=19 per group). In order to maintain the blind for participants, site staff, IRs, and sponsor staff, a central electronic database will be utilized for randomization based on validated computer-generated lists. The Stage 1 randomization list will utilize blocks to balance treatment assignments. Stage 2 randomization will utilize multiple validated randomization lists that re-randomize participants in a blinded manner to one of two new treatment assignments in a 1:1 ratio, excluding the previously assigned Stage 1 dose and the placebo condition as possibilities.

The blind may be broken for an individual participant if there is a clinically or medically urgent emergency requiring knowledge of the participant's marijuana condition assignment. This emergency unblinding would require approval from the site CI and Coordinating Investigator. For this purpose, the Randomization Monitor, who is designated to be unblinded for the study, will provide dose assignment through the electronic randomization system. Randomization information is only available within the web-based randomization system and only viewable by designated unblinded personnel. In all other cases, the blind will be maintained until all participants have completed the study and the database is locked. The Coordinating Investigator, Co-investigator, CI, IR, and participant will be blind to condition assignment.

Prior to enrollment, participants at Site 01 will be tracked with a screening number assigned sequentially starting at "S1001." Participants who meet eligibility criteria will be enrolled in the study and assigned a five-digit participant number. The first two digits will be "01" to identify the study site. The next three digits identify the participant within the site and will be assigned sequentially, with 101 corresponding to the first participant enrolled, e.g. the first participant enrolled at Site 01 will be 01101, second 01102, etc.

4.3 Recruitment and Participant Population

Candidates for participation will be 76 veterans over 18 years of age with PTSD who have served in the US armed forces with duration of PTSD symptoms lasting at least six months. A participant would not be excluded for having more than one traumatic event, a diagnosis of PTSD is not required to be related to military service based on the CAPS-5 assessment. Participants may be persons of any gender, race, or ethnicity. Participants will be recruited via letters of referral sent to psychiatrists and psychotherapists, contact with veterans' organizations, advertisements or announcements placed in appropriate locations or on appropriate internet sites and the sponsor website, and word of mouth.

Study volunteers will be interviewed by telephone to learn if they meet initial screening criteria according to an IRB-approved phone script. If the prospective participant is eligible and interested in taking part in the study based on the telephone assessments, qualified site staff will continue phone prescreening and will provide them with consent materials for review and consideration. If, after review, an applicant remains eligible and interested in taking part in the study, an appointment will be made to obtain informed consent to participate and for a face-to-face screening assessment to further evaluate eligibility to participate. If the prospective participant is not eligible or interested in taking part in the study based on the telephone assessments and do not complete the consent process, documentation on eligibility information will be destroyed at the end of the study, apart from the summary reason for ineligibility, which will be retained on the Screening Log.

Participants must be able to give adequate informed consent and be able to attend all required visits. Participants are required to pass an initial urine drug screen that is negative for opiates, benzodiazepines, cocaine, methamphetamines, THC, and amphetamines (unless prescribed), at the start of screening and agree to adhere to a minimum of two weeks abstinence from marijuana use prior to randomization. Cessation during the final two weeks of screening will be confirmed by pre and post blood/urine cannabinoid tests (methods described in Section 5.2.3 "Screen 2") [77]. Participants must agree to use only the marijuana provided to them by the study team while they are participating in the study and to not use any marijuana during the screening and two study washout periods. Participants must meet all study eligibility criteria by the end of the two-week screening period in order to be eligible for enrollment.

4.3.1 Participant Inclusion Criteria

Eligible individuals based on initial screening criteria must:

1. Meet DSM-5 criteria for PTSD with symptoms of at least six months duration.
2. Have PTSD of at least moderate severity (CAPS-5 score of 25 or more) at the time of baseline assessment.
3. Be a military veteran with PTSD.
4. Be at least 18 years old.
5. Be willing to commit to medication dosing and delivery method, to completing evaluation instruments, and attending all study visits.
6. Agree to use only marijuana provided by site staff and agree to required cessation periods for the duration of the study.
7. Report no current hazardous marijuana use, as defined by a score of < 8 on the CUDIT-R at time of screening, and completely abstain from marijuana during the 2-week baseline assessment period (biochemically verified via urine and/or blood cannabinoid concentrations).
8. Agree to video record all marijuana administrations and provide video to the site staff for review during study participation.
9. Agree to keep all marijuana provided by site staff securely stored in the provided lock box and not to share/distribute marijuana to any other individual.
10. Be stable on any pre-study medications and/or psychotherapy prior to study entry, agree to inform physician(s)/clinician(s) providing current care about participation in the study, and agree to report any changes in medication or psychotherapy treatment regimen during the study, to study staff.
11. If of childbearing potential, agree to use an effective form of birth control during study participation and may only be allowed to enroll and continue in the study based on a negative pregnancy test. Adequate birth control methods include intrauterine device (IUD), injected or implanted hormonal methods, abstinence, oral hormones plus a barrier contraception or double barrier contraception. Two forms of contraception are required with any barrier method or oral hormones (i.e. condom + diaphragm, condom or diaphragm + spermicide, oral hormonal contraceptives + spermicide or condom). Not of childbearing potential is defined as permanent sterilization, postmenopausal, or assigned male at birth.
12. Be proficient in reading and writing in English and able to effectively communicate with site staff.
13. Agree not to participate in any other interventional clinical trials during the study.

4.3.2 Participant Exclusion Criteria

Individuals who meet any of the following criteria will be excluded from participation:

1. Are pregnant or nursing, or are of child bearing potential who are not practicing an effective means of birth control.
2. Current or past DSM-5 diagnosis of dissociative identity disorder, eating disorder with active purging, personality disorders, primary psychotic disorder, bipolar affective disorder type 1, positive family history (first degree relative) of psychotic disorder or bipolar affective disorder type 1.
3. Have any allergies to marijuana or contraindication for smoking of marijuana.
4. Would present a serious suicide risk as assessed by the investigators, or who investigators believe are likely to require psychiatric hospitalization during the course of the study.
5. Current diagnosis or evidence of significant or uncontrolled hematological, endocrine, cerebrovascular, cardiovascular, coronary, pulmonary, gastrointestinal, immunocompromising, or neurological disease.
6. During the 2-week screening period, have a positive urine drug screen for opiates, methamphetamine, cocaine, and amphetamines (unless prescribed); meets DSM-5 criteria for a current substance use disorder (other than marijuana, caffeine or nicotine).
7. Meets criteria for current cannabis use disorder of moderate severity (4 or more of 11 DSM-5 criteria) and continued marijuana use during two 2-week screening period confirmed by quantitative blood and urine testing.
8. Are not able to give adequate informed consent.
9. Are not able to attend face-to-face visits or those who plan to move out of the area within the treatment period.
10. Have any current problem, which in the opinion of the investigators or medical monitor, might interfere with participation in the study.

5.0 Methods

5.1 Assessments and Measures

5.1.1 Outcome Measures

Primary Outcome Measure:

The Clinician-Administered PTSD Scale (CAPS-5) [78] is a semi-structured clinical interview administered by a blinded IR and is used to assess index history of DSM-5-defined traumatic event exposure [79], including the most distressing event, time since exposure, and total number of exposures, as well as frequency and severity of posttraumatic stress symptoms, as evidenced by CAPS-5 total score. The CAPS-5 provides diagnostic status (presence versus absence) of PTSD as well as PTSD symptom severity. Changes in the CAPS-5 total score will be used as the primary PTSD outcome measure, and will be the primary determinant of effect size for this pilot study. The IR will administer the CAPS at visits described in the Time and Events table either in person

or via telemedicine. The last month CAPS will be administered at Screen 2, Visit 5 and Visit 12. The last week CAPS will be administered at Visits 7 and 14. IRs will receive training on administering this measure from a research reliable trainer. Interviews will be recorded in as many instances as necessary to establish reliability of a random selection of interviews for accuracy. The CAPS-5 will be administered by trained interviewers throughout the study, who will be supervised by qualified personnel.

Secondary Outcome Measures:

The PTSD Checklist (PCL-5) with LEC plus criterion A (at baseline only) [80] is a 20-item self-report questionnaire in which respondents indicate the presence and severity of PTSD symptoms, derived from the DSM-five symptoms of PTSD [79]. Participants indicate how much distress they have experienced due to symptoms such as "Repeated, disturbing memories, thoughts, or images of a stressful experience from the past," "Trouble remembering important parts of a stressful experience from the past," and "Feeling irritable or having angry outbursts" on a five-point Likert-type scale (1=*not at all*, 5=*extremely*). The total PCL-5 score (a sum of all 20 items) provides an index of overall PTSD symptom severity.

The Inventory of Depression and Anxiety (IDAS) [81] is a 64-item self-report measure of depression and anxiety symptoms. Factor analytic research indicates that the IDAS has strong convergent and discriminate validity, as well as criterion validity [82]. Additionally, factor analytic research indicates that the general depression and anxiety subscales of the IDAS differentiate anxiety from depression [81, 82].

The Insomnia Severity Index (ISI) is a brief self-report measure of insomnia. It consists of seven questions, with responses made on a five-point Likert scale [83, 84]. Three items address difficulty at sleep onset, maintaining sleep and early waking, and four questions address perceived quality of sleep and effects of sleep difficulties on daily function. Questions are summed into a total score that ranges from 0 to 28 and can be interpreted as ranging from no signs of insomnia to severe insomnia. The ISI exhibits adequate to very good validity when compared with other self-report measures of sleep quality, statements concerning sleep quality and polysomnography, and is sensitive to changes in sleep quality [84-86].

Actigraphy (ACT) with ActiSleep and Monitor Actigraph devices will be used to measure objective sleep at baseline and all follow-up assessments. This noninvasive objective assessment device provides accurate and reliable measurements of sleep and wake including the following: amount of sleep, number and duration of awakenings, sleep efficiency, amount and intensity of physical activity, energy and activity, and sleep position.

The Inventory of Psychosocial Functioning (IPF) [87] is an 80-item measure that that was developed for use among individuals with PTSD. It assesses current psychosocial functioning across seven domains: romantic relationships, family, work, friendships,

parenting, education, and self-care. Summation of scores across domains yields a total score for psychosocial functioning. The IPF has excellent psychometric properties [87].

5.1.2 Safety Measures

The Time-Line Follow-Back (TLFB) method will be used to track medical and non-medical drug/substance use [88, 89]. A retroactive TLFB assessment will be conducted to record substance use during the 90 days prior to the intake assessment during screening. Participants will also be asked to assign monetary value to one gram of cannabis.

The Cannabis Use Disorders Identification Test-Revised (CUDIT-R) [90] is an eight-item self-report questionnaire used to assess problematic marijuana use within the past 6 months. Questions assess consumption (frequency), marijuana abuse (e.g., use in hazardous situations, inability to fulfill obligations), marijuana dependence (e.g., not able to stop using, spending a lot of time obtaining, using, or recovering from use), and negative consequences of use (e.g., problems with memory or concentration). Responses are summed, thus providing a continuous score of problematic marijuana use. The CUDIT has demonstrated high internal consistency and acceptable test-retest reliability, high specificity and sensitivity, as well as satisfactory predictive power [90].

The Marijuana Withdrawal Checklist (MWC) [91, 92] will be used to assess the presence of marijuana withdrawal symptoms during prior periods of abstinence. The MWC will be labeled the Behavior Checklist to minimize expectancy effects. It lists 32 symptoms for which participants indicate severity on a four-point scale (0=not at all, 1=mild, 2=moderate, 3=severe). The symptom list comprises the valid items found in prior marijuana withdrawal studies and additional non-specific items to minimize response bias. During the intake assessment, participants will be instructed to indicate whether and to what degree they experienced each symptom during past periods of marijuana abstinence lasting at least 48 hours, if they are current marijuana users. Instructions will be slightly different during the study than during the intake assessment. Participants will be asked to rate how they have felt since last completing the questionnaires. Administered in this way, the MWC has been used effectively to detect reliable marijuana withdrawal effects in several prior studies [91-96]. A total withdrawal discomfort score (WDS) will be computed from the Checklist and will be the outcome variable for this measure. This summary score will include the symptoms reliably observed in prior marijuana withdrawal studies [97].

The Columbia Suicide Severity Rating Scale (C-SSRS) is a clinician-administered measure of suicidal behavior devised to detect potential suicidal thoughts or behaviors during a clinical trial [97]. It consists of a “Baseline” form that assesses lifetime suicidal ideation, intensity, and behavior, and a form for assessing current suicidal ideation and intensity. The C-SSRS consists of a series of questions, and can be administered via face-to-face interview or over the telephone. The C-SSRS is a detailed interview, but the full interview is needed only if the initial screening questions about suicidal ideation and behavior are positive. The screening questions should be completed for every participant according to the Time and Events Table and as needed if the participant is showing any

signs of being suicidal. For management of suicidal thoughts, see Section 7.3 Risk Mitigation.

Site staff will assess general wellbeing (GWB) during each face-to-face visit and phone contact. Ratings on current demeanor and state of mind are collected on a scale of 1 “Very Stable and Calm” to 6 “Very Distressed.” Ratings on likelihood of deterioration are collected from A “Does Not Face Risk” to C “Faces Risk.” Results of this scale are intended to maintain participant safety throughout the study and will not be analyzed.

The subjective ratings of cannabis intoxication will be determined using the Drug Effect Questionnaire (DEQ). The individual items of the DEQ, 15 total, include ratings of drug effects (i.e. high, drug effect, good effect, bad effect), craving, and behavioral/mood states often associated with marijuana intoxication (i.e. relaxed, paranoid, hungry/have munchies, anxiety, irritability, feelings of heart racing/ pounding in chest). Participants will rate each item using a visual analog scale (VAS) anchored with “not at all” at one end and “extremely” at the other end. VAS measures have been reliably shown to be sensitive to detecting dose effects of a variety of drug classes including smoked marijuana. An analysis across studies assessing response to the DEQ found items to assess unique factors, with each item moderately to strongly related to others [98]. The DEQ has been used in studies assessing the effects of d-amphetamine [99] and MDMA [100]. This analysis also detected an association between “liking” and positive mood effects.

Participant vital signs, including blood pressure (systolic/diastolic), heart rate and body temperature, will be assessed at baseline and during each weekly visit, as described in the Time and Events table. Vital signs will be measured before, during, and after self-administration during introductory sessions. In addition, pulse oximetry will be assessed at baseline and before, during, and after introductory sessions.

Urine dipstick testing will detect the alcohol metabolite Ethyl Glucuronide (EtG). EtG is detectable in human urine for up to 80 hours after alcohol consumption. This will be performed to monitor recent alcohol consumption in study participants and confirm participant self-reports at each face-to-face visit.

The SCID-5-RV [101] is a structured clinical interview that assesses the dichotomous diagnoses (with indicators of severity) of psychological disorders, including substance use disorders. The SCID-5-RV has excellent psychometric properties and is a valid and reliable diagnostic tool [101]. The SCID for personality disorders (SCID-PD) will be used to assess for Axis 2 personality disorders. Staff will receive training on administering these measures from a research reliable trainer. Interviews will be recorded in as many instances as necessary to establish reliability of a random selection of interviews for accuracy. These assessments will be administered at baseline by trained interviewers throughout the study, who will be supervised by qualified personnel.

Dissociative Disorders Interview Schedule for DSM-5 (DDIS): Questions on the DDIS specifically addressing dissociative identity disorder symptoms (items 117-130) will be asked by the IR designee during Screening. These questions are part of an intensive

interview that includes questions concerning somatic and psychiatric symptoms. The interview is intended to assess and potentially distinguish between dissociative disorders and other disorders and between DID and a dissociative disorder not otherwise specified [102]. Owing to overlap with items found between the DDIS and other screening measures, only questions specifically addressing DID will be assessed.

The STOP-Bang Questionnaire (Chung et al., 2013) will be used as a self-report screening assessment for obstructive sleep apnea. Scores on the STOP-Bang will be collected to inform whether study participants are at high risk for sleep-related breathing disorders and will be considered as a possible exploratory baseline covariate in analysis of sleep continuity outcomes (e.g. Actigraphy).

AEs, including spontaneously reported reactions, will be collected as described in Section 8.0 of the protocol. Concomitant medications and therapies will be collected as described in Section 9.0 of the protocol.

5.1.3 Process Measures

The Daily Diary is an instrument developed by the sponsor to track potential AEs and assess daily use of study marijuana as well as other substances. The Daily Diary will be an electronic data capture form completed on an electronic device. Information collected with this diary will include participants' self reported quality and duration of sleep, the daily amount of marijuana used concomitant use of alcohol, medications, or other drugs, as well as potential AEs. Daily diary reports will be confirmed and verified by phone contact, reviewing video of participants' self-administration of marijuana, and qualitative urine drug testing at the site.

Laboratory testing of blood/urine cannabinoids will be conducted by an accredited laboratory with an appropriately sensitive, specific and validated assay, as specified in Section 10.2. Peripheral blood samples will be collected and stored for biomarker analysis of cannabinoids (THC, 11-OH-THC, THC-COOH), CBD metabolites and inflammation markers with appropriately sensitive, specific and validated assays for CRP, IL-1β, and IL-6. Peripheral blood will be drawn at specified times in the Time and Events Labs table and analyzed according to procedures described in Section 10.0.

Questions regarding the belief of condition assignment and certainty of the belief will be asked of site staff and participants at the End of Stage 1 and End of Stage 2. These beliefs are collected as a part of the sponsor's ongoing initiative to optimize the double-blind as a part of drug development studies.

Table 1: Time and Events (Administrative Procedures)

				**Stage 1**			**Cessation 1**		**Stage 2**			**Cessation 2**		**Stage 3**
	**Screen**	**Baseline**		**Introductory Sessions**	**Self-administration**	**Primary Endpoint**	**Cessation 1**	**Stage 2 Baseline**	**Introductory Sessions**	**Self-administration**	**Secondary Endpoint**	**Cessation 2**	**Final Outcome**	**Stage 3 (optional)**
**Visit #**	Prescreen & Screen 1	Screen 2	Enrollment	V1 & V2	V3 & V4	V5	V6	V7	V8 & V9	V10 & V11	V12	V13	V14	V15-V23 (as needed)
**Type of Visit**	Telemed Visit & Site Visit	Telemed Visit & Site Visit	Site Visit	Site Visit	Weekly Site Visits	Telemed Visit	Site Visit	Telemed Visit & Site Visit	Site Visit	Weekly Site Visits	Telemed Visit	Site Visit	Telemed Visit & Site Visit	Weekly Site Visits (as needed)
**Visit Timing**	Prescreen calls up to 2 months prior, in-person and Telemed procedures at least 2 weeks prior to Enrollment	2 weeks after Screen 1	2 weeks after Screen 1, once cannabinoid results are obtained or eligibility is determined (Day 0 begins at Enrollment)	Occur on 2 consecutive days (Week 1 begins following Intro Session 2)	Between start of Week 2 & start of Week 3	Between end of Week 3 & start of Week 4<br><br>May be combined with V6	Within 2 days of Primary Endpoint	Between end of Week 5 & start of Week 6	Post cannabinoid results Occur on 2 consecutive days (Week 6 begins after Intro Session 4)	Between start of Week 7 & start of Week 8	Between end of Week 8 & start of Week 9<br><br>May be combined with V13	Within 2 days of Secondary Endpoint	Between end of Week 10 & start of Week 11	Post cannabinoid results Week 11-18
**Phone Screen**	✓[A]													
**ICF**	✓													
**Brief Medical & Psych History**	✓													
**Directed Physical**	✓													
**Collect Therapy & Medications**	✓		✓	✓	✓		✓	✓	✓	✓		✓	✓	✓
**Enrollment & Randomization**			✓											
**Training on Self-Administration**				✓					✓					
**Video Data Review**					Weekly					Weekly				Weekly
**Dispense Drug**				Post V2	Weekly				Post V9	Weekly				Weekly
**Weigh Unused Marijuana**					Weekly		✓			Weekly		✓		Weekly
**Phone Calls**					Weekly[B]		Weekly[C]			Weekly[B]		Weekly[C]		Weekly[D]
**Collect AEs**			✓	✓	✓		✓	✓	✓	✓		✓	✓	✓

A = Initial phone screen with coordinator, secondary phone screen to be performed by CI/Qualified designee or Clinician. B = Participants will have 3 days of phone contact after each second introductory session in Stage 1 and Stage 2. (more contact may occur as needed) Participants will have a mid-week phone contact during Stage 1 and Stage 2 self-administration weeks 2 and 3. C = Participants will be contacted one week after the beginning of Cessation. D = Participants will have a mid-week phone contact.

Table 2: Time and Events (Laboratory Procedures)

				**Stage 1**			**Cessation 1**		**Stage 2**			**Cessation 2**		**Stage 3**
	**Screen**	**Baseline**		**Introductory Sessions**	**Self-administration**	**Primary Endpoint**	**Cessation 1**	**Stage 2 Baseline**	**Introductory Sessions**	**Self-administration**	**Secondary Endpoint**	**Cessation 2**	**Final Outcome**	**Stage 3 (optional)**
**Visit #**	Prescreen & Screen 1	Screen 2	Enrollment	V1 & V2	V3 & V4	V5	V6	V7	V8 & V9	V10 & V11	V12	V13	V14	V15-V23 (as needed)
**Type of Visit**	Telemed Visit & Site Visit	Telemed Visit & Site Visit	Site Visit	Site Visit	Weekly Site Visits	Telemed Visit	Site Visit	Telemed Visit & Site Visit	Site Visit	Weekly Site Visits	Telemed Visit	Site Visit	Telemed Visit & Site Visit	Weekly Site Visits (as needed)
**Visit Timing**	Prescreen calls up to 2 months prior, in-person and Telemed procedures at least 2 weeks prior to Enrollment	2 weeks after Screen 1	2 weeks after Screen 1, once cannabinoid results are obtained or eligibility is determined (Day 0 begins at Enrollment)	Occur on 2 consecutive days (Week 1 begins following Introductory Session 2)	Between start of Week 2 & start of Week 3	Between end of Week 3 & start of Week 4	Within 2 days of Primary Endpoint	Between end of Week 5 & start of Week 6	Post cannabinoid results Occur on 2 consecutive days (Week 6 begins after Introductory Session 4)	Between start of Week 7 & start of Week 8	Between end of Week 8 & start of Week 9	Within 2 days of Secondary Endpoint	Between end of Week 10 & start of Week 11	Post cannabinoid results Week 11-18
**Blood Cannabinoid**	✓[A]	✓[A]					✓[A]	✓[A]				✓[A]	✓[A]	
**Biomarker**		✓					✓	✓				✓	✓	
**Qualitative Urinary Drug**	✓	✓		✓	✓		✓	✓	✓	✓		✓	✓	✓
**Quantitative Urinary Drug**	✓[A]	✓[A]					✓[A]	✓[A]				✓[A]	✓[A]	
**Urinary EtG**	✓	✓	✓	✓	✓		✓		✓	✓		✓	✓	✓
**Urinary Pregnancy**	✓	✓		✓	✓		✓	✓	✓	✓		✓	✓	✓
**Clinical Labs**	✓													
**ECG**	✓													
**BP & BT**	✓			✓[C]	Weekly		✓		✓[C]	Weekly		✓	✓	Weekly
**Pulse Oximetry**	✓			✓[B]					✓[B]					

A = Quantitative Urine THC testing to be conducted in THC positive participants only. Blood THC testing only if indicated. Blood and urine will be obtained and stored regardless of THC results.
B = Completed before & immediately after self-administration and every 30 minutes thereafter until end of the visit.
C = BP and BT will be obtained prior to self-administration, 30 minutes after starting self-administration and again at the end of each session, approximately four hours after starting self-administration.

Table 3: Time and Events (Study Measures)

				Stage 1			Cessation 1		Stage 2			Cessation 2		Stage 3
	**Screen**	**Baseline**		**Introductory Sessions**	**Self-administration**	**Primary Endpoint**	**Cessation 1**	**Stage 2 Baseline**	**Introductory Sessions**	**Self-administration**	**Secondary Endpoint**	**Cessation 2**	**Final Outcome**	**Stage 3 (optional)**
**Visit #**	Prescreen & Screen 1	Screen 2	Enrollment	V1 & V2	V3 & V4	V5	V6	V7	V8 & V9	V10 & V11	V12	V13	V14	V15-V23 (as needed)
**Type of Visit**	Telemed Visit & Site Visit	Telemed Visit & Site Visit	Site Visit	Site Visit	Weekly Site Visits	Telemed Visit	Site Visit	Telemed Visit & Site Visit	Site Visit	Weekly Site Visits	Telemed Visit	Site Visit	Telemed Visit & Site Visit	Weekly Site Visits (as needed)
**SCID**	✓													
**CAPS-5**		✓				✓		✓[F]			✓		✓[F]	
**IDAS**			✓		✓		✓	✓		✓		✓	✓	
**ISI**			✓		✓		✓	✓		✓		✓	✓	
**IPF[G]**			✓				✓	✓				✓	✓	
**PCL-5[E]**	✓		✓		✓		✓	✓		✓		✓	✓	
**ACT**		✓[C]		✓[C]	✓		✓	✓	✓	✓		✓	✓	
**MWC[H]**	✓		✓				✓	✓				✓	✓	
**CUDIT-R**	✓												✓	
**DEQ**				✓[A]					✓[A]					
**Daily Diary**			✓	✓[B]	✓[B]	✓	✓	✓	✓[B]	✓[B]	✓	✓	✓	✓[B]
**C-SSRS & GWB**	✓		✓	✓	✓		✓	✓	✓	✓		✓	✓	✓
**STOP-Bang**	✓													
**Belief of Condition**							✓					✓		
**TLFB**	✓													
**DDIS**	✓													

A = Completed before & immediately after self-administration and every 30 minutes thereafter until end of session.
B = Completed immediately after each self-administration
C = Actigraphy use must begin one week prior to the first Introductory Session.
D = Final week only.
E = Will measure past week. LEC plus Criterion A to be assessed at baseline only.
F = CAPS-5 assessments at these timepoints will measure the last week. All others will measure the last month.
G = Visit 6 will be based on past 3 weeks, Visit 7 will be based on the past 2 weeks, Visit 13 will be based on the past 3 weeks, Visit 14 will be based on the past 2 weeks.
H = Will measure the past 2 weeks.

Figure 1: Summary of Events

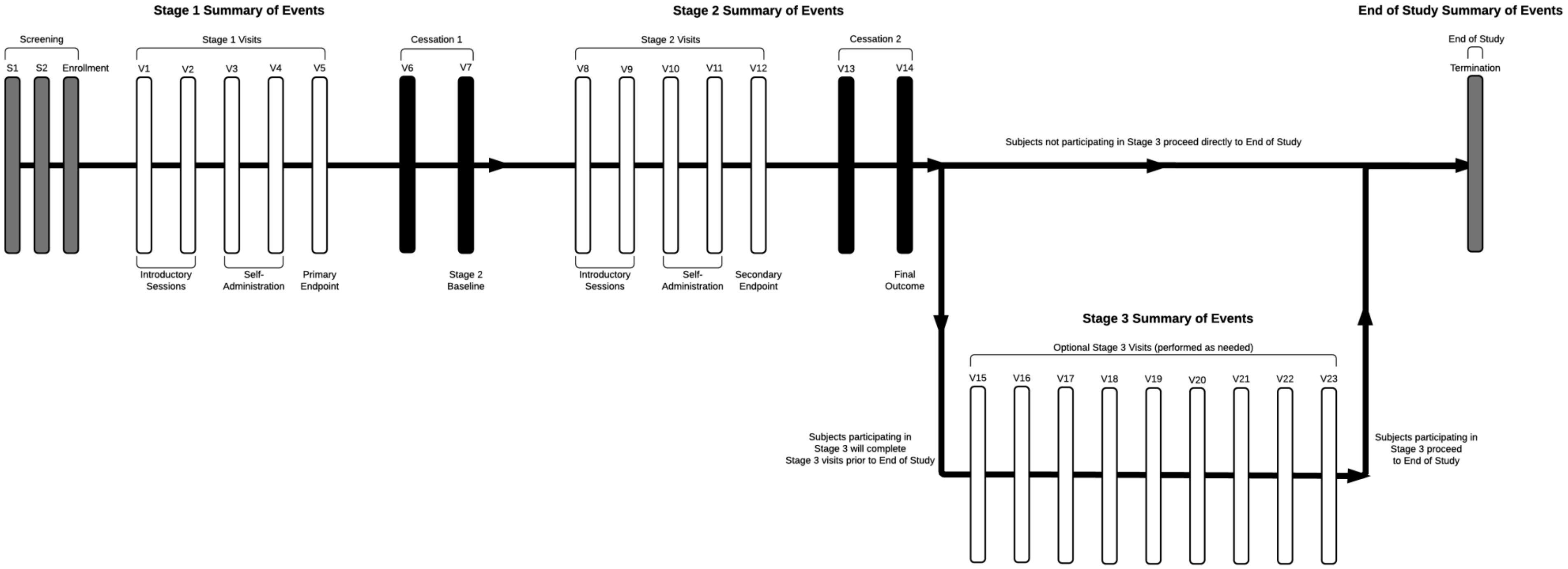

5.2 Study Procedures and Visit Descriptions

5.2.1 Pre-screening

All individuals who enter pre-screening, as defined in this section, should be assigned a screening number and recorded on the "Screening Log". The candidate's screening number will also be noted on the signed informed consent form (ICF). Candidates who do not meet all eligibility criteria will not be enrolled. An IRB-approved phone screening script will be used to initially screen candidates. Pre-screening information will be retained for candidates who provide specific permission via the ICF for utilization and retention of this information. If candidates do not provide this permission, only the reason for not enrolling will be retained in study records. A Case Report Form (CRF) will not be completed for candidates who are not enrolled. These candidates will be documented only on the screening log and source records completed during screening. The site staff should record either the reason why an individual was not enrolled or the enrollment date and assigned participant number on this log. It is the responsibility of the investigator to file this document in the investigator site file (ISF) to be readily available for on-site monitoring and/or for inspection by the relevant authorities.

5.2.2 Screen 1

If pre-screening information after telephone screening with site staff and the CI or qualified designee suggests the candidate may be suitable for the study, site staff will schedule the Screen 1 visit. The Screen 1 visit consists of an in-person site visit and an IR visit in person or by telemedicine or phone.

a. Explain and obtain written informed consent from the candidate. Written informed consent must be obtained prior to performing any study-specific tests or evaluations.
b. Ensure that the candidate has been issued a screening number. Complete the Screening Log for this visit.
c. The candidate will provide a medical and psychological history including current and past medications and therapies. Prior to enrollment the subject should sign a medical release form to allow the CI to review medical records to ensure the participant has no underlying medical conditions.
d. Review with participants of childbearing potential their ability to become pregnant and commitment to practice appropriate birth control as determined by the CI or qualified designee for the total duration of the study.
e. The CI or qualified designee will assess prior substance use through the TLFB procedure for the past three months and ask the participant to assign a monetary value to one gram of cannabis.
f. The CI or qualified designee will assess GWB.
g. The CI or or qualified designee will administer the C-SSRS to assess suicide risk.
h. The candidate will complete the PCL-5 with LEC-5, MWC, CUDIT-R, and STOP-Bang.
i. The IR will perform all relevant portions of the Structured Clinical Interview for DSM-5 Diagnoses – Research Version (SCID-RV) and SCID for personality

disorders, which are module-based structured psychiatric interviews, to assess study eligibility. To establish reliability, the IR will have the option to video record the SCID interviews in as many instances as necessary.

j. The IR designee will administer the relevant four questions of the Dissociative Disorders Interview Schedule for DSM-5 (DDIS).

k. A physician, who may be the CI or qualified site staff, will perform a medical history directed physical examination. The examination will involve the following procedures:
   - Blood pressure
   - Pulse
   - Pulse Oximetry
   - Height/weight
   - Body temperature
   - Examination of head, eyes, ears, nose, throat, skin, heart, lungs, abdomen and extremities
   - Brief neurological exam (cranial nerves 2-12, sensory, motor, reflexes and cerebellar function)

l. The following clinical assessments will be collected and reviewed by the CI or qualified site staff:
   - Electrocardiogram (ECG), the ECG will be used to establish eligibility and any clinically significant findings would be captured in the Medical History.
   - Additional blood and urine testing will include serum electrolytes, metabolic profile, HIV screen, urinalysis, and complete blood count. Results of HIV serology will be kept confidential and appropriate referral for counseling may be necessary in accordance with state law. The clinical laboratory values will not be captured in the CRF, but will be used to establish eligibility and will be kept with the participant’s source record. Clinically significant findings would be captured in the Medical History.
   - Qualitative urine drug screening
   - Qualitative urine-dip EtG test to assess alcohol consumption
   - Urine-dip pregnancy test in participants with childbearing potential
   - Blood cannabinoid analysis will be collected (will be sent for analysis only if quantitative urine analysis is inconclusive in determining abstinence)
   - Quantitative urine drug testing (will send for analysis only if pre- and post-cessation qualitative urine drug screens are positive for THC-COOH)

m. If, upon examination, there are questions raised about possible medical problems, the CI will request a review of medical records and request additional tests or assessments as indicated.

n. The site staff will discuss having participant identify a community observer who can be contacted to participate in the study. This is a person who the participant typically interacts with on at least a weekly basis (e.g. friend, family member, co-worker, counselor/therapist) and who can comment on their health, behavior, and psychosocial engagement. The participant will provide the site staff with a telephone number in order to reach the community observer for the WBR. The site staff will obtain the observer’s informed consent via written signature.

o. Participants will be instructed to not use any non-study cannabis products by any

route of administration for the duration of the treatment period, beginning at this visit.

p. Participants will be trained in Grounding and will be issued a Grounding Handout. Grounding involves participants detaching themselves from emotional pain by focusing on the outside world rather than what's going on inside of them. It is useful for extreme emotional pain.
q. Participants will be instructed in diaphragmatic breathing.
r. If the candidate meets all initial screening criteria, site staff will schedule the Screen 2 Visit.

5.2.3 Screen 2

The Screen 2 visit should be completed approximately two weeks after Screen 1 and consists of an in-person site visit and an IR visit by telemedicine or phone.

a. A blinded IR will administer the last month CAPS-5 in a face-to-face visit or via telemedicine.
b. To establish reliability, the IR will have the option to video record the CAPS interview in as many instances as necessary.
c. Peripheral blood will be collected for Blood Cannabinoid testing and Biomarker analyses.
d. Participants will be required to stop using non-study marijuana for a 2-week baseline screening period (and for the duration of the treatment period). Compliance will be assessed by either: 1) Qualitatively negative urine drug screen, or 2) Sequential quantitative urine drug screens that indicate 90% or greater confidence in abstinence based on the algorithm developed by Huestis and colleagues [77], or 3) 80% confidence in compliance based on the Huestis algorithm and ratios of THC to 11-OH-THC and THC-COOH in blood that suggest no recent use has occurred. If a participant fails the test for abstinence, they will be given one additional chance at the 2-week baseline abstinence period.
e. Perform a urine-dip pregnancy test on participants of childbearing potential.
f. Perform a urine-dip EtG test on all participants to assess alcohol consumption.
g. Participants will be issued and receive orientation on use of the Actigraph. The Actigraph should be used for one week prior to the first Introductory Session. Participant will be instructed to wear the Actigraph 24 hours a day, removing only when there is potential for water damage (e.g. dishwashing or showering). Participants will be issued the Actigraphy/iPad use agreement at this visit.

5.2.4 Enrollment

Day 0 of the study will be the day of enrollment. Enrollment will be completed after Screen 1 and 2, one week of wearing the Actigraph, quantitative cannabinoid results are available from applicable participants and after eligibility can be established.

Enrollment can be combined with Introductory Session 1.

a. Inquire about any possible changes in the participant's health to ensure they continue to meet eligibility criteria.
b. After eligibility is confirmed, the candidate may be enrolled. Issue a participant number.
c. The CI or site staff will schedule introductory sessions and the beginning of the period of active dosing.
d. Collect information on any changes in psychotherapy or medications for the participant.
e. Participants will receive a research participant identification card ("wallet card") stating that they are a research participant and may test positive for drugs and listing CI contact information, the telephone number for a 24-hour hotline for participant support, and instructions on how and when to present the card.
f. Obtain/confirm the name and telephone number of an emergency contact from the participant for use throughout the study.
g. The participant will complete the IDAS, ISI, IPF, PCL-5, MWC, and Daily Diary.
h. The CI or qualified site staff will administer the C-SSRS and GWB.
i. Perform a urine-dip EtG test on all participants to assess alcohol consumption.
j. Randomization will occur after enrollment and prior to the day of the first introductory session.
k. Throughout the study, concomitant medications and therapies and AEs will be collected at each contact as described in Sections 8.0 and 9.0 of the protocol.

5.2.5 Introductory Sessions in Stage 1 (Visit 1 and Visit 2) and Stage 2 (Visit 8 and Visit 9)

This visit will be conducted approximately one week after the Screen 2 visit, after the Actigraph has been worn for one week, after eligibility has been established, and for applicable participants, after quantitative cannabinoid results are available. To standardize method of self-administration and to assess psychological risk, this protocol will include two four-hour long introductory sessions, where the participants will self-administer marijuana under observation and receive training, on two consecutive days as an added safety procedure prior to the initiation of each treatment phase. A CI will be on-call during Introductory Sessions by phone or in-person.

a. At the first introductory session, the participant will bring their Actigraph to the study site. The site staff will copy all recording files, note the time and date stamp and save for baseline analysis. The Actigraph memory will be cleared, the device charged and returned to the participant to use for the self-administration period.
b. Site staff will provide the participant with information about marijuana, including expected psychoactive and physical effects, and a standardized procedure for smoking marijuana using the pipe provided for use throughout the study. Methods will be discussed for documenting and managing any side effects of smoking marijuana. This will include information about community support, breathing strategies, as well as what rescue psychotherapy or medications are available for

participants to use if needed.

c. Participants will complete a qualitative urine drug test and a pregnancy test (if of childbearing potential).
d. Perform a urine-dip EtG test on all participants to assess alcohol consumption.
e. On the first of the two introductory sessions, participants will self-administer at least one puff of marijuana using the provided marijuana pipe. The pipe will contain a maximum of 0.08 g of marijuana. Participants will be allowed to self-administer additional puffs if desired, but no more than the 0.08 g in the pipe. Participants will wait 2 to 5 minutes between puffs. If more than 5 minutes passes, the administration portion of the Introductory Session will cease. This method of self-administration will provide a standardized minimum and maximum level of initial exposure across participants. 0.08 g was chosen to calculate a maximum 10 mg dose of THC if the participant receives the high THC product.
f. On the second session, participants will be allowed to self-administer marijuana in an *ad libitum* manner for up to 45 minutes from the first inhalation to simulate outpatient use. After this point, the measurements to be taken immediately after smoking will be obtained.
g. In both sessions, pulse oximetry and DEQ assessments will be conducted before, immediately after, and then every 30 minutes for four hours post-marijuana exposure.
h. In both sessions, C-SSRS and GWB will be collected before self-administration and four hours after self-administration.
i. Vital signs (blood pressure/body temperature) will be obtained at the beginning of each introductory session prior to self-administration, 30 minutes after starting self-administration and again at the end of each session, approximately four hours after starting self-administration.
j. Participants will be oriented on the use of a lockable storage box and a portable tablet device.
k. The site staff will instruct the participants on electronic diary entry completion.
l. Participants will either arrange a ride from the study site to their current place of residence or the site staff will assist them in finding a means of transport from the study site.
m. Participants will not be provided marijuana to take home between Introductory Session 1 and Session 2.
n. The introductory visits will be conducted again at the beginning of Stage 2 because the type of marijuana provided to participants will be different, and participants randomized to placebo in Stage 1 will be receiving active marijuana for the first time in Stage 2.
o. At the end of the second introductory session in the absence of contraindications, site staff will provide the participant with a weekly kit containing seven packages of 1.8 grams of marijuana for participants to self-administer for one week of the three-week self-administration period. The type of marijuana provided will change from Stage 1 to Stage 2, but will not change within stages. Participants will receive clear instructions to not use marijuana not provided to them as part of the study and not to share the study marijuana with any other individual. The participant will be provided with an Instructional Handout at the first Introductory

Session.

5.2.6 Daily Contact after Introductory Sessions

a. Starting after the second introductory session, the CI or site staff will contact the participant via telephone or in person on a daily basis for three days. Additional contact may be made as participants or clinicians feel necessary.
b. The daily telephone contact is expected to last between 5 and 15 minutes, or as long as necessary to address any participant's concerns and to assess the participant's wellbeing.
c. Collect information on any changes in psychotherapy, AEs, or medications for the participant.
d. The CI or qualified designee will administer the C-SSRS.
e. Site staff will record GWB and responses to questions on any changes in mood or behavior after each telephone call. If the CI or qualified designee has any concerns about emerging psychiatric symptoms, then he or she will refer the participant to the site CI for evaluation.
f. After the first week, participants will return to the study site for another week's worth of marijuana.

5.2.7 Self Administration During Stage 1 and Stage 2

Participants will self-administer marijuana for three weeks during Stage 1 and Stage 2. Participants will come to the study site for weekly visits. Weekly visits may be completed within three days of when they are scheduled. If any visits are completed out of window, the CI and sponsor should assess effects on protocol compliance on a case-by-case basis. Week 1 starts on the day after Introductory Session 2. If a participant returns on the inside of their visit window, they will be allowed to keep their unused jars until the next visit to complete their previous week of self-administration.

a. During each three week self-administration period, there will be a daily upper quantity limit of 1.8 grams per day, which participants may use at any time that day, with a day defined as a period of 24 hours in this study, from 12:00 AM (midnight) to 11:59 PM of that day. There will be no lower quantity limit, and participants do not have to use any of the daily allotment should they decide that symptoms do not require medication on any particular day.
b. Participants will be required to video record each self-administration on the same portable tablet. The participants will be instructed on reducing risks of others viewing or publicly presenting their recordings on the Internet.
c. The Daily Diary will be completed electronically though a portable tablet immediately after each self-administration.
d. Participants will be required to wear the Actigraph daily.
e. The CI or site staff will telephone the participant midweek to assess GWB and any AEs.
f. The participant will undergo weekly assessments at the study site as described below.

5.2.8 Weekly Evaluations During Stage 1 (Visit 3 and Visit 4) and Stage 2 (Visit 10 and Visit 11)

Participants will meet with the CI or site staff at the study site once a week during the self-administration period.

a. Inquire about any possible changes in the participant's health to ensure they continue to meet eligibility criteria.
b. Collect information on any changes in psychotherapy, AEs or medications for the participant.
c. Obtain vital signs from the participant.
d. Obtain a urine sample to test drugs of abuse.
e. Participants of childbearing potential will have a pregnancy test performed.
f. The CI or qualified site staff will administer the C-SSRS.
g. The participant will complete the IDAS, ISI, and the PCL-5.
h. Participants will return any unused marijuana in the original package dated by day of use from the last week of self-administration. Any unused marijuana from each weekly allotment will be returned to the CI or designee to be weighed and accounted for at the end of the week prior to receiving the next weekly supply. Marijuana will be labeled with the participant number and date. The remaining marijuana will be preserved and securely stored at the site until destruction following DEA regulation.
i. The participant will bring their portable tablet to the study site. The site staff will review all video recording files, noting the time and date stamp. Study staff will verify participant identity, safety, and compliance through review of video recordings, as well as telephone contact reports. The portable tablet will be cleared of content and returned to the participant.
j. The participant will bring their Actigraph to the study site. The site staff will copy all recording files, note the time and date stamp and save for analysis. The Actigraph memory will be cleared, the device charged and returned to the participant to use for the next week. Upon analysis, if any days are missing from recordings, participants will be re-trained the next time they are contacted by site staff.
k. If video data review indicates compliance with the protocol, the site staff will provide the participant with a week of daily marijuana for take-home use and will remind the participant to complete the Daily Diary.
l. Perform a urine-dip EtG test on all participants to assess alcohol consumption.
m. Identical weekly evaluations like this will occur until the next cessation period.

5.2.9 Primary and Secondary Endpoint Evaluations in Stage 1 (Visit 5) and Stage 2 (Visit 12)

This visit will be conducted at the end of self-administration in each stage. This meeting will take place in person, via telemedicine or phone. This visit may take place on the same day as Visit 6 or Visit 13 (Day 1 of cessation in Stage 1 or Stage 2).

a. Participants will meet with the IR for a 60 to 90 minute evaluation.
b. The IR will administer the last month CAPS-5.

5.2.10 Two Weeks of Abstinence from Marijuana Self-Administration during Cessation 1 (Visit 6) and Cessation 2 (Visit 13)

Participants will cease to self-administer marijuana for a two-week interval. After a face-to-face visit, participants will complete a telephone contact and self-report measures one week after the start of cessation. The Daily Diary will continue to be completed daily.

a. At the start of the Cessation period, immediately after completion of the primary/secondary endpoint assessment, participants will visit the study site. This visit may be conducted on the same day as the endpoint assessment or within two days of it.
b. The participant will complete the IDAS, ISI, IPF, PCL-5 and the MWC.
c. The CI or qualified designee will administer the C-SSRS and GWB.
d. The participant and a blinded site staff member will provide their belief of condition assignment.
e. Inquire about any possible changes in the participant's health to ensure they continue to meet eligibility criteria.
f. Collect information on any changes in psychotherapy, health or medications for the participant.
g. Obtain vital signs from the participant.
h. Obtain a urine sample to test drugs of abuse. Participants of childbearing potential will have a pregnancy test performed.
i. Participants will return any unused marijuana in the original package dated by day of use from the last week of self-administration. Any unused marijuana from each weekly allotment will be returned to the CI or designee to be weighed and accounted for. Marijuana will be labeled with the participant number and date. The remaining marijuana will be preserved and securely stored at the site until destruction per DEA instruction.
j. Peripheral blood will be collected for Blood Cannabinoid analyses and Biomarker analyses.
k. The participant will bring their portable tablet to the study site. The site staff will review all video recording files, noting the time and date. Upon receipt, site staff will verify participant identity, safety, and compliance through review of video recordings, as well as telephone contact reports. Upon review, if any days are missing from recordings, participants will be re-trained by site staff. The portable device will be cleared of content and returned to the participant.

l. The participant will bring their Actigraph to the study site. The site staff will copy all recording files, note the time and date stamp and save for analysis. The Actigraph memory will be cleared, the device charged and returned to the participant to use for the next week. Upon analysis, if any days are missing from recordings, participants will be re-trained the next time they are contacted by site staff.
m. The site staff will instruct the participant to complete the Daily Diary to assess, sleep patterns, continuing substance and medication use patterns and AEs during cessation.
n. The site staff and participant will discuss the two-week period of cessation of marijuana use.
o. One week after the beginning of cessation, participants will complete the MWC , IDAS, ISI, IPF, and PCL-5 electronically. Participants will be reminded to complete self-report measures in a timely manner.

5.2.11 Re-Baseline Evaluation at End of Cessation 1 (Visit 7) and Cessation 2 Final Outcome (Visit 14)

At the end of the Cessation period, two weeks after completion of the primary/secondary endpoint assessment, participants will visit the study site.

a. The participant will complete the IDAS, ISI, IPF (past 2 weeks), PCL-5 and the MWC.
b. The participant will complete the CUDIT-R only at Visit 14.
c. The CI or qualified site staff will administer the C-SSRS and GWB.
d. Participants will meet with the IR for a 60 to 90 minute evaluation, either in a face-to-face or telemedicine meeting to administer the last week CAPS-5.
e. During the site visit, site staff will inquire about any possible changes in the participant’s health to ensure they continue to meet eligibility criteria.
f. Collect information on any changes in psychotherapy, AEs or medications for the participant.
g. Obtain vital signs from the participant.
h. Obtain a urine sample to test drugs of abuse.
i. Participants of childbearing potential will have a pregnancy test performed.
j. The participant will bring their Actigraph to the study site. The site staff will copy all recording files, note the time and date stamp and save for analysis and return to the participant in Stage 1 and Stage 2. Participants will not be given the Actigraph in Stage 3.
k. The site staff will instruct the participant to complete the Daily Diary during upcoming self-administration through electronic patient reported outcomes (ePRO).
l. Perform a urine-dip EtG test on all participants to assess alcohol consumption.
m. Peripheral blood will be collected for Blood Cannabinoid levels analysis and Biomarker analyses.
n. After this assessment, participants will either continue in the crossover arm (Stage 2) with associated introductory sessions as described in Section 5.2.3, or they may

continue to the optional Stage 3 as described in Section 5.2.9 if they have already completed Stage 2. Participants who do not continue to Stage 3 will be required to return the lockable storage box and portable tablet to site staff, and will exit the study after this visit.

5.2.12 Stage 3

Stage 3 is an optional continuation of the study for participants who request the amount of returned marijuana from Stage 1 or Stage 2 to be issued from a new supply of marijuana. Site visits will be conducted weekly as needed to complete Stage 3, which will last up to two months or until remaining marijuana is consumed.

a. The CI will dispense marijuana in the amount that was left over from Stage 1 or Stage 2 according to the participants randomization in Stage 1 and/or Stage 2 from a new supply if the participant requests to take part in Stage 3. Participants will be able to choose to receive a weekly kit containing the same blend of marijuana from a new supply as their Stage 1 or Stage 2 study product supply at each weekly visit.
b. Participants will retain the locked storage box and portable tablet to be used for Daily Diary completion as in Stage 1 and Stage 2 if they chose to participate in Stage 3.
c. Participants will be required to video record each self-administration on the same portable tablet.
d. The CI or site staff will telephone the participant midweek to assess GWB and any AEs.
e. The participant will undergo weekly assessments at the study site as described below.

During each weekly visit:

a. Inquire about any possible changes in the participant's health to ensure they continue to meet eligibility criteria.
b. Collect information on any changes in psychotherapy, AEs or medications for the participant.
c. Obtain vital signs from the participant.
d. Obtain a urine sample to test drugs of abuse. Participants of childbearing potential will have a pregnancy test performed.
e. Perform a urine-dip EtG test on all participants to assess alcohol consumption.
f. The CI or qualified site staff will administer the C-SSRS.
g. Continued weekly visits will end when the participant has used an amount equivalent to the amount of returned marijuana or does not wish to continue using study marijuana, or two months have passed, at which point he or she must return all remaining marijuana to the study site. The CI or site staff will weigh any returned marijuana. The marijuana will be stored securely at the site for drug accountability.
h. The participant will return the portable tablet and locked box to the site staff.
i. After completing their allotted Stage 3 visits, participants will exit the study.

5.3 Removal of Enrolled Participants from the Study

Participants can withdraw consent at any time without prejudice. The CI can withdraw a participant if, in his or her clinical judgment, it is in the best interest of the participant or if the participant cannot comply with the experimental procedures and related visits that are critical for safety, and this will be recorded in the participant's source records and CRF. Participants who are not able to comply with required periods of abstinence from marijuana use will be withdrawn from treatment. If the CI withdraws a participant from treatment, the CI will explain the reason for withdrawing to the participant, and refer them to standard clinical care for PTSD. Participants who are withdrawn will not be denied care within or outside of the VA or participating institutions. Participants will be clinically monitored after withdrawal by the CI. Whenever possible, the tests and evaluations listed for the termination and outcome visits will be carried out. Efforts will be made to obtain information about AE resolutions, if applicable. All participants who sign informed consent , meet study eligibility criteria including 2 weeks abstinence period prior to randomization, and who receive initial dose of study medication will be included in intent to treat analyses (including data/outcome data for study participants who withdraw consent up to the time consent is withdrawn).

Participants who discontinue treatment during the study will not be replaced. Recruitment and enrollment will continue until 76 participants have completed the Primary Endpoint.

5.4 Premature Discontinuation of the Study

The sponsor or the CI (following consultation with the sponsor) have the right to discontinue this study at any time. If the trial is prematurely terminated, the CI is to promptly inform the study participants and will arrange appropriate follow-up. If the trial or study is prematurely discontinued, all procedures and requirements pertaining to the archiving of documents will be observed. All other study materials will be returned to the sponsor, and will be treated in accordance with federal and local regulations.

6.0 Investigational Product

6.1 Substance Description and Activity Related to Proposed Action

The active material to be used in this protocol is dried plant material from the cannabis plant (marijuana). *Cannabis* refers to the genus within the *cannabacae* family, containing possibly two species *c. sativa* and *c. indica*. They are hardy annual flowering plants, and dioecious, meaning there are male and female plants [103]. This plant or extracts from this plant have been used medicinally for thousands of years, and were legal in the US until 1937. Marijuana was removed from the United States Pharmacopeia and National Formulary [104]. Currently, it is a Schedule 1 controlled substance, meaning that it is illegal to possess, sell or distribute marijuana outside of research studies under federal laws. Individual states within the US have significantly reduced penalties for possessing small quantities of marijuana, have legalized marijuana, or have laws or legislation

permitting physicians to recommend medical use of marijuana. Marijuana contains several active compounds, with THC acting as the chief psychoactive compound. THC acts on cannabinoid CB1 receptors in the brain, as well as CB2 receptors in the periphery, particularly within the immune system. These recently discovered receptors are activated by at least three endocannabinoids and endogenous fatty acids related to arachidonic acid. CB1 receptors are found throughout the human brain. CBD may also contribute to the psychoactive profile of marijuana [65, 105, 106], and possess therapeutic potential [106, 107]. The subjective and potentially therapeutic actions of marijuana may be directly or indirectly influenced by other compounds found in the plant [106].

The marijuana to be used in this study will be provided at cost by NIDA, after receipt of all the required regulatory approvals. Four types of marijuana obtained through the NIDA Drug Supply Program that vary in ratios of THC to CBD will be used: High THC, High CBD, THC/CBD, or placebo. Concentrations of 7-15% will be considered "High" and <2% will be considered "Low," subject to availability of marijuana grown and harvested by NIDA. *Ad libitum* self-administration will be permitted up to a maximum of 1.8 grams/day.

The doses of marijuana (excluding placebo) were chosen because they contain a range of THC and CBD ratios and potencies generalizable to what many veterans are currently using to manage PTSD symptoms in non-clinical settings in states with legalized medical marijuana. The final doses and concentration to be tested are subject to availability through the NIDA Drug Supply Program. Prior to the study start, laboratory testing of the marijuana will be completed to verify chemical composition of each concentration group. The active doses are expected to produce all of the commonly reported subjective effects of marijuana. The physiological effects are expected to be tolerable based on previous observational studies in the literature. The placebo is expected to produce minimal side effects, without the range of psychological and subjective effects seen in active doses.

6.2 Substance Concentrations, Packaging and Labeling

This protocol will compare marijuana differing in THC or CBD concentrations. Any unused portions of the marijuana that participants did not consume during each day will be placed back in the daily packet and kit from which it came and collected on a weekly basis, with all packages labeled with day of use returned to the CI at the end of the self-administration in Stage 1 and 2. Participants may choose to receive the amount of marijuana returned in Stage 1 or Stage 2 as a part of Stage 3. Any unused marijuana will be tracked during the study and stored for drug accountability until destruction per DEA recommendations.

6.2.1 Doses

Study participants will be assigned to receive one of four marijuana conditions based on cannabinoid content: ~12.40% THC/~0.03%CBD (High THC), ~0.53% THC/~13.94% CBD (High CBD), ~7-9% THC/~7-9% CBD (THC/CBD) or ~0% THC/~0% CBD

(placebo). Marijuana will be self-administered by the participant through smoking the study marijuana.

Participants will be introduced to the marijuana concentration and the self-administration method to be used during introductory sessions during which they will smoke the study material. The CI or qualified designee, under the direction of a Schedule 1 license holder, will supply participants with packages containing seven doses of 1.8 mg per day for self-administration daily during a three-week period. Any daily unused portions will be placed in the packaging for that day and labeled with the date of use, with all unused material returned to the CI or designee during each weekly evaluation during the period of self-administration, and at the conclusion of the three-week period of active dosing, for precise weighing. The CI or designee will store each participant's unused study drug in a DEA approved secured container until destruction.

Table 4: Participant Distribution by Concentration and Stage

**Stage 1: N=76 (up to 116) Participants Randomized 1:1:1:1 to the Following Four Dose Groups**				
**Stage 1 Arms**	High THC	High CBD	THC/CBD	Placebo

**Stage 2: Participants from Stage 1 are Re-randomized 1:1 to the Following Dose Groups**				
**Stage 1 Arms**	**Stage 2**			
**High THC**		High CBD	THC/CBD	
**High CBD**	High THC		THC/CBD	
**THC/CBD**	High THC	High CBD		
**Placebo**	High THC	High CBD		

Table 5: Marijuana Doses in Stage 1

**Dose**	**Min Number of Participants Receiving Dose**
High THC/Low CBD	19
Low THC/High CBD	19
High THC/High CBD	19
Low THC/Low CBD	19

Table 6: Marijuana Doses in Stage 2

**Dose**	**Approximate Number of Participants Receiving Dose***
High THC/Low CBD	25
Low THC/High CBD	25
High THC/High CBD	26

* Due to enrollment and dropouts to achieve N=76 for primary endpoint, the actual number of Stage 2 participants may be higher or lower

6.2.2 Packaging

Marijuana will be sent in bulk from the NIDA to the study site. The marijuana will be received and packaged by appropriately qualified site personnel per the Schedule 1 license. There will be a designated unblinded witness observing all marijuana packaging following site SOPs and Sponsor requirements. The qualified individuals will package all marijuana in blinded daily packets. Daily packets for weekly self-administration will be placed in blinded kits. Stage 3 kits will be packaged according to study needs upon subject requests based on returned Stage 1 or Stage 2 marijuana.

6.2.3 Labeling

Each concentration of marijuana will come in large bags labeled by NIDA. Unblinded site staff, under observation by a designated unblinded witness, will package 1.8 grams of bulk marijuana in separate packages following site SOPs for weekly kits. Each blinded kit label will include the protocol number, substance name, kit number, sponsor name, a place for participant number to be entered, and a statement that the material is for self-administration limited by law to investigational use only. Additional labels may be required per site SOPs. Marijuana will be individually packaged for the first introductory session to allow for use of 0.08 grams. The second introductory session will be packaged in the amount of 1.8 grams. Marijuana concentration will match condition assignment for each participant in each stage according to the Randomization List. Each blinded packet label will include the protocol number, substance name, kit number, packet number (1-7), sponsor name, a place for participant number to be entered, a place for date of use to be entered, and a statement that the material is for self-administration only in clinical trials. Blinded labels will be provided by the sponsor and applied by qualified individuals at the site. The package labels will not contain any information about the concentration of the marijuana in order to assure blinding of participant and CI. A record linking the kit number to the concentration of the marijuana provided to that participant will be maintained as part of pharmacy records.

Figure 2: Drug Package Labels

Kit Label	Daily Packet Label
MAPS Study# MJP1<br>Investigational Product: Cannabis (marijuana)<br>Dose: Blinded<br>Kit #: XXXX<br>Participant Number _____________<br>Keep in the lock box<br>Store in a cool location. Keep out of heat<br>Self-Administer as per protocol<br>Caution-Limited by Law to Investigational Use Only	MAPS Study# MJP1<br>Investigational Product: Cannabis (marijuana)<br>Dose: Blinded<br>Kit #: XXXX<br>Packet #: X<br>Participant Number _____________<br>Date of use: ___/___/____<br>Keep in the lock box<br>Store in a cool location. Keep out of heat<br>Self-Administer as per protocol<br>Caution-Limited by Law to Investigational Use Only

6.3 Substance Accountability

Forms will be provided to the site to track drug accountability and administration throughout the study. Equivalent forms of the sites choosing may be used. Blinded drug accountability will be reviewed during routine monitoring visits. Records pertaining to the use of Schedule 1 compounds will be maintained in accordance with relevant Federal and State Regulations, and institutional policies, where applicable. Marijuana dispensed during the study will be tracked by individual kit number, packet number, date, and participant number.

6.4 Substance Storage and Handling

Cannabis (marijuana) is a Schedule 1 controlled substance and will be stored and handled in compliance with relevant institutional policies (if applicable), Federal and State

regulations and in accordance with Drug Enforcement Administration (DEA) requirements.

6.5 Substance Stability

Information on the concentrations of THC and CBD, and stability of these compounds, will be provided by NIDA. The sponsor may obtain an additional analysis from an independent testing laboratory with required DEA Schedule 1 license registration.

7.0 Risks of Participation

7.1 Risks of Screening, Study Procedures, Assessments and Measures

Blood draws and a full medical examination are required to establish eligibility for the study. Blood draws are also required to assess quantitative cannabinoid levels during marijuana self-administration and during cessation of use as well as biomarkers for effects on inflammation. Temporary discomfort, inflammation or infection could arise as a result of sampling blood at the punctured vein. Measures of blood pressure, body temperature, and heart rate will be taken to assess drug effects and participant safety. Participants may experience mild discomfort from having blood pressure assessed. Submitting to a full medical examination may also cause discomfort or psychological distress. Since medical examinations and blood draws are required to establish eligibility for the study, they cannot be omitted from the protocol. Psychological assessments will be obtained through interviews. Because these interviews require individuals to discuss their condition, they may prove upsetting for some. Because psychiatric interviews and discussion of PTSD symptoms are used during screening, they cannot be avoided. The CI has experience working with people with PTSD, and they will seek to reduce anxiety and distress during these interviews.

A screen lock and pin will always be used on the tablet and encryption will be used to minimize the likelihood that anyone without the computer passcode could access stored files on the hard drive. Recording marijuana use with video is necessary to verify that the participant is using the marijuana as directed, and to assure that the participant is not diverting any of the marijuana. The Sponsor will enable the ability to delete files from the tablet computer remotely in the case that a device is lost or stolen. Breach of confidentiality may still happen even with these measures, another individual may be able to view recordings of participant marijuana use during self-administration should they obtain the participant's portable tablet. If this happens, it is possible that such recordings could be uploaded to a computer or displayed on a social media website. A breech may also happen through mishandling of participant records. This risk is mitigated by the use of standard research procedures for the secure storage and management of participant PHI described in more detail in Section 7.3.

As with any medical procedure, there are potential risks associated with the use of telemedicine. In rare cases, information transmitted may not be sufficient (e.g. poor resolution or choppy video connection) to allow for appropriate medical decision making by site staff. Delays in medical evaluation and treatment could occur due to deficiencies or failures of the equipment or internet service. Though the telemedicine system that will be used is HIPAA compliant and secure, in very rare instances, security protocols could fail, causing a breach of privacy of personal medical information.

7.2 Risks of Self-administering Marijuana

There is an extensive literature on the risks of habitual marijuana use in humans, and a sizeable but considerably smaller literature on the acute effects of marijuana, including AEs. Most risks associated with ingesting or inhaling marijuana relate to its psychoactive effects, though marijuana can also produce acute effects on the cardiovascular system and continued use can produce effects on the pulmonary system. Psychoactive and acute cardiovascular effects are transient and dissipate after the effects of the substance have waned.

Marijuana can alter mood, affect, and perception, producing increases in positive and negative mood states (as euphoria and anxiety), and intensifying sensory experiences, such as music seeming more intense [108]. Marijuana can impair attention, memory, and visual tracking, and slow psychomotor performance. In a review of the literature, subjective effects were more strongly associated with marijuana than other effects [109]. One study found impaired visual tracking in male occasional cannabis users approximately an hour after administration [110]. The combination of alcohol and marijuana impaired simulated driving, particularly night-time driving, with regular cannabis users making a greater number of errors than occasional users [111], and the same team of researchers reported that alcohol and marijuana affected performance on a field sobriety test more than either substance alone [112]. Marijuana may provoke psychotic symptoms or psychosis in vulnerable individuals, though marijuana use alone is not a “cause” of psychosis [113, 114]. The literature is inconclusive, but indicates an association between regular marijuana use and psychosis or exacerbated psychotic symptoms.

Regular, heavy use of marijuana is associated with impairments in cognitive function, especially in the area of short-term memory and executive functioning, with impairment retained up to a week after abstaining from use, but no longer detectable after 28 days of abstinence [115-117]. Visual tracking and divided attention was impaired in heavy daily cannabis users compared with ecstasy-user controls [118], but tracking and attention improved with three weeks of abstinence from marijuana. Since marijuana interferes with attention, alters mood and may generate shifts in sensory attention and perception, it is not surprising that regular use may be taxing on cognitive task performance. The degree of potential impairment experienced after a month of daily use cannot be estimated from these findings. It is likely that if impaired cognitive function is present, it will not remain after prolonged abstinence [119].

Marijuana use may be associated with increased occurrence of panic attacks. A prospective (longitudinal) study in adolescents reported that marijuana use or dependence was associated with a greater likelihood of experiencing panic attacks[120], and people with marijuana dependence were more likely to report experiencing panic attacks [121].

Van Ours and colleagues (2013) examined the predictive value of marijuana use onset and monthly use upon self-reported suicidal ideation through examining data from a longitudinal study of a cohort born in 1972, concluding that onset of marijuana use preceded likelihood of reporting suicidal ideation in males, but not females [122]. The authors did not find that suicidal ideation preceded marijuana use, though it is notable that questions concerning suicidal ideation were not posed until respondents were 15 years old. Previous research has addressed suicidal ideation in marijuana users, largely in samples of adolescents.

Though a review of studies found that marijuana impairs most skills used in driving motor vehicles, driving and simulation studies fail to find strong effects of marijuana upon driving [123]. Researchers conducting controlled studies of people driving under the influence of marijuana reported that effects, while present, were relatively small and comparable to other medicines or alcohol [124]. This may be the result of people under the influence of marijuana overestimating their level of impairment, and thus driving more conservatively [123, 125]. Nonetheless, epidemiological studies of road accidents have found a relation between use of marijuana, including blood THC levels, and road accidents [126-129], with higher levels of THC associated with greater impairment in driving. Elvik (2012) carried out a meta-analysis of illicit psychoactive drug use and the likelihood of a road traffic accident [127], reporting that cannabis was associated with minor, but not significantly, increased odds of traffic accidents - lower than the rate associated with benzodiazepines. Sewell and colleagues concluded that a review of the literature suggests a degree of inter-individual variability in degree of impairment experienced by drivers after marijuana, including less impairment in experienced users [123]. They recommend that people not drive or use heavy machinery for up to three hours after marijuana use, and that people using marijuana seek a designated driver. Other recent analyses support Sewell and colleagues' recommendation that people restrict driving for three hours after marijuana use [130, 131].

Like many substances that produce increased positive mood and relaxation, marijuana can lead to abuse or dependence in some people, with approximately 1.6% of the general population experiencing dependence upon marijuana [132]. The rate at which people who try marijuana become dependent is estimated as either slightly lower than or similar to that for alcohol, and higher than rates of dependence for hallucinogenic (psychedelic) compounds [133].

Acutely, marijuana increases heart rate, increases supine blood pressure, and, after higher doses, produces orthostatic hypotension; it increases cardiac output, decreases peripheral vascular resistance, and dose-dependently decreases maximum exercise performance. Changes in cardiovascular function may occur with prolonged use. These include:

hypotension when lying down, an increase in blood volume, slowed heart rate and diminished circulatory response to exercise [134]. These findings are in line with findings in animals of enhanced parasympathetic activity. After reviewing the literature, Jones (2002) concluded that the cardiovascular effects of marijuana posed little risk to young, healthy adults, while increased cardiac work, increased hypotension, and increased catecholamines might pose greater risk for older adults [134].

A case report detailed an instance of a type of heart attack [ST-segment elevation myocardial infarction (MI)] in a 37-year old man who reported smoking marijuana prior to arrival at the emergency department [135], possibly as a result of having an inadequate myocardial oxygen supply from smoking marijuana. Cardiac events associated with marijuana have been reported since 1979 [136], and a previous ST-segment elevation MI was reported in 2010 [137]. Researchers presented a case series addressing marijuana use and stroke [138]. The existence and nature of the relationship between marijuana use and stroke remains controversial [139].

Regular and heavy marijuana use is associated with increased symptoms of chronic bronchitis, coughing, production of sputum, and wheezing [140, 141]. Regular marijuana use may impair function of alveolar macrophages, a type of immune cell found in the lung [141, 142]. Reduced alveolar macrophages could place individuals at increased risk of lung infection. One of three studies of lung function in people reporting regular, and often heavy, use of smoked marijuana failed to find a reduction in lung function, and another found reduced lung function but concluded that this was related to confounding factors [140, 143, 144]. A review of literature addressing marijuana use and lung injury concluded that findings were often inconsistent [145]. Marijuana use does not appear to be associated with lung cancer [145-147]. Rather, the positive association between extended periods of marijuana use and lung cancer may be related to other confounding factors, such as co-occurring tobacco use. Duration of use in the studies reviewed by Hashibe and colleagues is considerably longer than the three-week periods occurring in this study [147].

There are several reports of cannabis-associated hyperemesis syndrome in regular marijuana users, marked by excessive, cyclical vomiting and abdominal pain that is relieved by taking a hot bath or shower [148-150].

The immunological effects of marijuana and cannabinoids are complex and largely appear to arise from effects on CB2 receptors rather than central CB1 receptors. Some of the benefits of marijuana, such as for multiple sclerosis, may relate in part to anti-inflammatory and immunosuppressive effects [151, 152]. However, marijuana and cannabinoids failed to affect immune function in HIV-positive individuals [153, 154]. Regular marijuana users have greater numbers of a cannabinoid receptor implicated in regulating immune function, the CB2 receptor, which is generally considered to have immunosuppressive and anti-inflammatory effects [155], and in vitro studies suggest that THC and marijuana may reduce immunosupportive Th1 cytokines and increase immunosuppressive Th2 cytokines [151, 156]. It is possible that marijuana may increase the risk of opportunistic infections. However, in studies of HIV-positive individuals using

either marijuana or oral THC (as dronabinol) at similar use levels to this study failed to find any changes in T-cell (CD4 or CD8) profiles, findings that do not support this form of immunosuppression [153, 157].

Participants who receive the placebo marijuana are exposed to combustion products without receiving either THC or CBD. They will also be less likely to experience the adverse effects associated with marijuana and THC described above, such as anxiety or impaired performance on tests of cognitive function. Participants will be permitted to remain on medications and therapies to allow for continued symptom management throughout the study.

Published reports of events associated with cannabis use include seizures upon cessation of use, pancreatitis, and gingival (gum) enlargement. In a report discussing the potentially anti-convulsant properties of cannabis, Hegde and colleagues (2012) discuss two cases where seizures increased in two patients after cessation of cannabis use and upon entry into an epilepsy unit [158]. Two case reports describe acute pancreatitis in marijuana users, with one report acknowledging the difficulty of linking the previous cannabis use with subsequent pancreatitis [159, 160]. According to Howaizi and colleagues (2012), only six cases of pancreatitis associated with marijuana have been reported in the literature [160]. A recent case report describes gingival enlargement in two extensive marijuana users [161].

Beyond these risks, there are only a few reports of adverse effects occurring outside the organs and systems listed above. There are no known effects on the liver (and only a few case reports of effects on the kidneys [108]).

Regular use of marijuana throughout pregnancy may have effects on birth weight, as well as specific tasks involving visual analysis or processing. However, to date, there are no reports of teratological effects from marijuana use [108, 162-165]. THC can pass into breast milk [165]. Participants of childbearing potential enrolled in this study will be required to use an effective method of birth control, and the study will exclude participants who are pregnant or lactating.

Using the study drug poses a social risk of actual or perceived stigmatization. Marijuana use is a socially stigmatized activity [166, 167]. Participants may be ostracized or experience social stigma from relatives, friends or community members who view or hear about their smoking marijuana, or they may be aware of and internalize these prejudices relating to marijuana use.

Participants may test positive after urinary assay for drugs of abuse, as those used for workplace drug testing, or as a result of police response to a traffic violation. Even participants who are not using their marijuana on a daily basis may test positive for marijuana for up to three weeks after study participation. Testing positive for marijuana could pose risk of arrest or job termination.

For further information, please view *Information for Health Care Professionals* [168] that has been submitted along with the protocol.

7.3 Risk Mitigation

Marijuana is associated with acute risks as well as risks of continued daily use. Chief amongst these are unwanted psychological effects, including anxiety or paranoia, cardiovascular and pulmonary effects, impaired driving, and abuse liability.

The CI will minimize risks by carefully screening participants for the presence of any contraindicating factors and by carefully preparing participants for the expected effects of marijuana. Contraindicating factors include presence or history of psychotic disorder, a positive family history in first-degree relative (parent or sibling) of psychotic disorder or bipolar affective disorder Type 1, cerebrovascular, cardiovascular or coronary conditions, and past or current substance abuse. Prior to receiving supplies of marijuana, study participants will be prepared for the effects of the substance during two extensive supervised introductory sessions described in Section 5.2.5. They will be informed of what to expect and they will have an opportunity to smoke the marijuana that they have been randomly assigned to receive in a standardized manner and in the presence of site staff. A CI will be on-call during Introductory Sessions by phone or in-person. Participants will be telephoned on a daily basis for the first three days following the second introductory session of Stage 1 and Stage 2, and telephone contact will be made once, midweek, during the next three weeks to assess participant GWB and to detect any new psychiatric symptoms distinct from PTSD.

The CI or qualified designee will administer the C-SSRS according to the Time and Events table and as needed depending on clinical presentation of the participant to monitor for development and severity of suicidal ideation or behavior. The clinician will follow the Standard Operating Procedure for management of suicidal participants to assess elevated or imminent risk using best clinical judgment.

If the Since Last Visit C-SSRS reveals current serious Suicidal Ideation (Scores of 4 or greater), indicating low risk, at the time of the assessment or positive Suicidal Behaviour (Scores of 1 or greater) then the participant will be referred appropriately for further management as described below.

1. If the participant has current suicidal ideation, but has no specific plan to commit suicide (Suicidal Ideation Score=4), the clinician administering the C-SSRS will:

    a. Recommend the participant call the National Suicide Prevention Lifeline at 1-800-273-8255 before leaving the laboratory.
    b. Participants would be required to speak with a study clinician for evaluation and appropriate course of action, and encouraged to contact their personal therapist, if they have one.
    c. Participants would be provided with a list of mental health resources.

d. If revealed prior to enrollment, these participants may not be enrolled according to the Exclusion criteria based on the clinical judgment of the CI.
e. If this finding is treatment-emergent, the participant may be removed from participation in the study based on clinical judgement of the CI or qualified designee. If these events emerge after enrollment and are clinically significant, they will be collected on the Adverse Events page. The participant's continuing eligibility will be assessed by the CI and the Medical Monitor.
f. Unless the steps above result in the participant leaving for psychiatric care right away or the clinician advises otherwise, the visit would be continued.
g. If it is determined that the participant is at imminent risk of suicide, the CI or CI-designated staff member will do one of the following: 1) Escort the participant to the Emergency Department; 2) Escort the participant to an appropriate mental health services facility (e.g. hospital psychiatric unit); or 3) call 911 and ensure that the participant is transferred to the responding medical personnel.

2. If the subject has suicidal ideation, and a plan to commit suicide (Suicidal Ideation Score=5), the clinician administering the C-SSRS will assess whether the risk is imminent. A score of 5 does not necessarily indicate an immediate risk if the thoughts are fleeting, fairly easily controlled, and deterrents are strong. If there is no imminent risk, the clinician will follow the procedure described in number 1. If there is imminent risk of suicidal behavior, the CI will:

   a. Call the National Suicide Prevention Lifeline at 1-800-273-8255 immediately.
   b. Participants would be required to speak with a study clinician for evaluation and appropriate course of action, and encouraged to contact their personal therapist, if they have one, prior to leaving the laboratory.
   c. If it is determined that the participant is at imminent risk of suicide, the CI or CI-designated staff member will do one of the following: 1) Escort the participant to the Emergency Department; 2) Escort the participant to an appropriate mental health services facility (e.g. hospital psychiatric unit); or 3) call 911 and ensure that the participant is transferred to the responding medical personnel.
   d. If the participant will not comply and wishes to leave without consultation, call 911. Explain that the participant is in immediate danger of committing suicide. Provide a complete description of the participant and give any other needed details to ensure the subject’s safety.
   e. Provide the subject with a list of mental health resources.
   f. Notify the study team as soon as possible. This includes all site staff and all study team members representing the Sponsor.
   g. If revealed during screening, these participants would not be enrolled according to the Exclusion criteria.
   h. If this finding is treatment-emergent, the participant may be removed from participation in the study based on clinical judgment of the CI or qualified

designee. If these events emerge after enrollment and are clinically significant, they will be collected on the Adverse Events page. The participant's continuing eligibility will be assessed by the CI and the Medical Monitor.

The CI will address a number of risks by enrolling participants without contraindicating conditions, including psychotic disorders and major medical conditions affecting the heart or lungs. Participants who pose a major suicide risk will not be enrolled in the study. Risks posed by cessation of marijuana use will be reduced by requiring a period of abstinence from marijuana upon the start of screening. Participants using marijuana before entering the study are required to abstain from use for two weeks before receiving study marijuana. Any participant that the CI or Medical Monitor believes has contraindicating history of, or current, moderate to severe substance use disorder will not be enrolled.

Untoward psychological reactions to marijuana will be dealt with by preparing participants for the subjective effects of the substance, and through first smoking marijuana in the presence of site staff during two introductory sessions. During this time, site staff will be able to help address any anxiety or paranoid feelings that may arise. A CI will be on-call during Introductory Sessions by phone or in-person.

Participants will be informed of the effects that marijuana might have on driving and they will be advised to avoid driving immediately after use by seeking a designated driver and by waiting a minimum of three hours after use prior to driving a motor vehicle. Participants will arrange rides home after each introductory session, and if they are unable to do so, the CI will assist them in locating a ride from the study site.

Potential reproductive risks will be mitigated by restricting enrollment to persons who are not pregnant or lactating, and by requiring that participants of childbearing potential use an effective form of birth control and weekly pregnancy testing.

All study participants will be issued a study participant identification card (e.g. “wallet card”) stating that they may test positive for drugs of abuse as a result of being a research participant, listing the number of a 24-hour hotline, and containing contact information for the CI, identifying the sponsor and the relevant IRB, and the study National Clinical Trial Registry (NCT) number. The CI will remind participants that they may still be cited or face penalties for erratic driving and the card will not mitigate this.

The CI will address risks to confidentiality arising from the existence of digital video recordings of participant marijuana use by instructing participants to store their cameras securely in the lockbox provided to them as part of the study and informing them of the risks of exposing their personal video data to the internet. If a study computer tablet is lost or stolen, the study team will remotely delete files from the device, notify local police in the case of theft, and make every attempt to recover the unit. All tablets will be password protected and encrypted to minimize the likelihood that misplaced units will result in breech of confidentiality. Given the safeguards in place, breech of confidentiality

from the tablet computer is unlikely as long as participants follow instructions for use and storage. All participant PHI will be stored in locked files or on secure computer servers, and managed in accordance with patient confidentiality procedures pertaining to electronic systems.

The CI will discuss with participants the perceived stigma they ascribe to marijuana use and to consider the degree to which friends, family or other people within the community might respond if they observe or learn about the participant’s marijuana use.

This study involves take-home doses of study marijuana. To discourage the risk of diversion of unused marijuana during Stages 1 and 2, participants can choose to receive the amount of unused marijuana returned during Stage 1 or Stage 2 in Stage 3.

7.4 Medical Emergencies

If a participant experiences a medical emergency during the introductory sessions, the CI will be on call to determine course of action. If a participant experiences a medical emergency during the self-administration period at a location other than the study site, he or she will be directed to call 911 and inform the CI upon stabilization.

In the event of a psychological crisis arising during the study, participants and their community observers will be instructed to call the Participant Hotline as soon as possible. This call will be routed to the CI if it is confirmed that there is indeed a crisis. The CI, or qualified designee if the CI is unavailable, will be on call at all times to receive information from the Participant Hotline.

Upon learning about the psychological crisis, the CI will assess the AE by:

- Obtaining a detailed history of the event over the phone
- Collecting information on any concomitant medications
- Verifying the above with the community observer if necessary
- Assessing expectedness, seriousness, severity, and relation to study drug
- All AEs will be assessed regardless of whether they occurred prior to or after self-administration.
- If the participant requires immediate care, they will be referred to the Emergency Department of the closest hospital.

The Mobile Crisis Response Team (if applicable), Emergency Department, Hospital, or Primary Care Provider, or the CI may prescribe medications or take steps to stabilize the participant experiencing an AE.

While participants are participating in Stage 1 of the study, which includes a placebo group, results of the THC drug test could break the study blind for a participant. During any discussion with a provider about how to treat a medical event, the CI or site staff may need to inform the provider of the participant's medical history and course of treatment in the study. The CI or site staff should inform the provider of the likelihood that the

participant will test positive for marijuana, and determine with the provider if the study blind must be broken in order to effectively treat the participant. The CI should discuss delaying routine drug screens administered during treatment unless it is medically necessary.

Regardless of condition assignment, all necessary steps will be taken to ensure participants receive whatever medical care is necessary for an AE. If an outside treatment provider informs the participant of the results of a drug test for marijuana during Stage 1 of a participant's study visits, the participant must be withdrawn from the study in order to protect the study blind.

8.0 Adverse Events

An AE is defined as any untoward or unfavorable medical occurrence in a clinical research study participant, including any abnormal sign (e.g. abnormal physical exam or laboratory finding), symptom, or disease, temporally associated with the participants' involvement in the research, whether or not considered related to participation in the research. This definition includes concurrent illnesses or injuries and exacerbation of pre-existing conditions.

An unexpected AE is one that is not listed in the current Investigator’s Brochure or an event that is by nature more specific or more severe than a listed event.
All AEs will be monitored by the CI or site staff until resolution or, if the AE becomes chronic, a cause identified. If an AE is unresolved at the conclusion of the protocol, a clinical assessment will be made by the CI and/or Medical Monitor as to whether continued follow-up of the AE is warranted.

The severity of events reported on the AE CRF will be determined by the CI as:

- Mild: no limitation in normal daily activity
- Moderate: some limitation in normal daily activity
- Severe: unable to perform normal daily activity

The relationship of the study treatment to an AE will be determined by the CI, based on the following definitions:

1. Not Related

The AE is not related if exposure to the investigational product has not occurred, or the occurrence of the AE is not reasonably related in time, or the AE is considered unlikely to be related to use of the investigational product, i.e. there are no facts (evidence) or arguments to suggest a causal relationship, or the AE is more likely related to the participant’s pre-existing condition.

2. Possibly Related

The administration of the investigational product and the AE are considered reasonably related in time and the AE could be explained by causes other than exposure to the investigational product.

3. Probably Related

Exposure to the investigational product and AE are reasonably related in time and the investigational product is more likely than other causes to be responsible for the AE, or is the most likely cause of the AE.

The relationship of the study treatment to an AE will be determined by the CI based on the assessment of the participants' report of symptoms.

8.1 Spontaneously Reported Reactions

Common expected AEs, that have been compiled from the literature on smoked marijuana reported in studies of human volunteers, are referred to as "spontaneously reported reactions" are: altered time perception, anxiety, difficulty concentrating, difficulty remembering things, dry mouth, feeling intoxicated (as drug effect, feeling high), feeling of heart racing/ or pounding in chest, feeling hungry, perceptual alteration (as enhanced sensation), sedation and feeling stimulated [105, 169]. Some participants may report feelings of paranoia or unusual thoughts [65]. AEs that are included in the spontaneously reported reactions list will be separated in data analysis for review of expected vs. unexpected AEs.

8.2 Serious Adverse Events

The Site CI or qualified designee is responsible for determining if an AE is serious or non-serious. An SAE is defined as any untoward medical occurrence that at any dose:

- Results in death
- Is life-threatening (i.e., the participant was, in the opinion of the investigator, at immediate risk of death from the event as it occurred); it does not refer to an event which hypothetically might have caused death if it were more severe
- Requires or prolongs inpatient hospitalization
- Results in persistent or significant disability/incapacity (i.e., the event causes a substantial disruption of a person's ability to conduct normal life functions)
- Results in a congenital anomaly/birth defect
- Requires intervention to prevent permanent impairment or damage
- Is an important and significant medical event that may not be immediately life-threatening or resulting in death or hospitalization, but based upon appropriate

medical judgment, may jeopardize the patient/participant or may require intervention to prevent one of the other outcomes listed above

AEs which do not fall into these categories are defined as non-serious. It should be noted that a severe AE need not be serious in nature and that a SAE need not, by definition, be severe.

In addition, a pre-existing event or condition that results in hospitalization should be recorded in the medical history. The hospitalization would not result in the event or condition being reported as a study-related SAE unless, in the view of the investigator, hospitalization was prolonged as a result of participation in the clinical trial or was necessary due to a worsening of the pre-existing condition. This is because the onset of the event (the reason for the procedure) occurred before the participant was entered into the trial. Hospitalization for cosmetic procedures, non-emergency prophylaxis, or abortion does not result in an SAE report unless, in the view of the investigator, hospitalization for these procedures was prolonged as a result of participation in the clinical trial.

8.3 Adverse Event Collection

AEs that will be collected during the protocol:

- SAEs will be collected through termination.
- All AEs will be collected from enrollment until end of the active study period (e.g. end of Cessation 2 or Stage 3, as applicable)
- Events related to planned treatments or physician visits for baseline conditions collected in the Medical History will not be collected unless there is an exacerbation of the condition.
- Any AE leading to withdrawal from the protocol will be collected throughout the study.
- All AEs related to changes in psychiatric status will be collected throughout the study.

AEs will be collected during introductory sessions and throughout Stage 1, Stage 2, and Stage 3, as well as associated cessation periods, on the Daily Diary. Information needed to determine severity and relatedness will be collected and confirmed by telephone contact conducted according to the Time & Events table. Each reported AE will be actively followed during follow-up phone calls or weekly visits until resolution. AEs will be recorded in the respective AE log eCRF based on onset date, e.g. an AE reported during Introductory Session 1 or the Stage 1 self-administration period would be recorded on the Stage 1 AE log eCRF. CI or qualified site staff are responsible for review of safety data.

8.4 Adverse Event Reporting

All SAEs which occur during the course of the trial, whether considered to be associated with the study drug or not, must be reported to the sponsor within 24 hours, or at the latest on the following working day, of the site CI learning that an event occurred, irrespective of causality:

- Contact information for the MAPS Medical Monitors and Study monitor are provided in a separate document.
- All SAEs are to be assessed regardless of whether they occurred after drug administration, comparator product, placebo, or no treatment.
- SAEs will be assessed by the site CI and Sponsor Medical Monitor(s) for relatedness, seriousness, and unexpectedness and reported regulatory agencies, IRB and CI according to applicable regulations and policies.
- CI should notify their IRB according to IRB-specific timelines promptly after assessment and communication with the sponsor.
- SAE reporting to competent authorities is done by an authorized delegate of MAPS.

The Medical Monitor(s) will provide the required medical expertise in conjunction with site medical personnel to address trial-related medical questions or problems. In this capacity, the Medical Monitor(s) are responsible for the overall safety of the participants, the ongoing safety evaluation of the investigational product and the notification of all concerned investigators/institutions of findings that could adversely affect the safety of participants.

The site CI will communicate as needed with the Medical Monitors and study monitor to determine if the SAE is subject to expedited reporting and what follow-up information is needed for evaluation. If no consensus can be reached, the different assessments and the reasons for this must be summarized in writing. In any case the most conservative assessment drives the reporting procedure. The time frames for reporting SAEs to regulatory authorities begins when the sponsor has “initial information” of the event regardless of the form that initial notification takes. Expedited timelines will be followed according to regulatory, institutional, and IRB requirements.

The blind may be broken for an individual participant if there is an AE or other emergency requiring knowledge of the participant's condition assignment. This emergency unblinding would require approval from the site CI and Coordinating Investigator. For this purpose, the Randomization Monitor will provide dose assignment through the electronic randomization system.

The Sponsor will collaborate with investigators to ensure that all reports are submitted to the appropriate agency and IRB/EC within the required time frame and that they contain accurate and current information. The sponsor is responsible for ongoing safety evaluation of the investigational product and for the prompt notification of investigators conducting a MAPS-sponsored study and regulatory authorities of findings that could

adversely affect the safety of participants, impact the conduct of the trial, or alter the IRB's approval/favorable opinion to continue the trial.

Such information may be distributed to investigators by means of periodically revised investigator brochures, reprints or published studies, reports or letters to clinical investigators, or other appropriate means. Any employee, contractor, or contract organization working on behalf of MAPS who discovers a serious adverse experience in the context of a clinical trial is responsible for alerting clinical trial staff. MAPS is responsible for collecting serious adverse experience information, submitting safety reports to regulatory agencies, and notifying investigators of safety issues.

9.0 Collection of Concomitant Medications and Therapies

Information about all concomitant prescription medications, and only over the counter (OTC) medications associated with an AE, will be collected from screening up to the day of the final study visit in Cessation 2 or Stage 3, as applicable, including reasons for use, date started, date ended and dose information if available. Medications recorded per protocol, including medications taken to treat AEs, will be recorded on a concomitant medications form eCRF. Information on participation in evidence-based psychotherapy for PTSD will be collected throughout the study. Therapies and healthcare utilization recorded per protocol will be recorded on a concomitant therapies form eCRF.

10.0 Laboratory Assessments

10.1 Clinical Laboratory Assessments

The CI or qualified study personnel will examine laboratory assessments gathered at screening, for assessing participant eligibility. The CI will use a list of normal ranges to conclude whether participants are eligible for the protocol, and will indicate justification for admitting participants with abnormal values.

The following laboratory assessments will be performed as specified in the Time and Events table.

Serum electrolytes and the metabolic profile, which includes:

- ALT/SGPT
- Albumin:globulin (A:G) ratio
- Albumin, serum
- Alkaline phosphatase, serum
- AST/SGOT
- Bilirubin, total
- BUN
- BUN:creatinine ratio
- Calcium, serum
- Carbon dioxide

- Chloride, serum
- Creatinine, serum
- Globulin, total
- Glucose, serum
- Potassium, serum
- Protein, total, serum
- Sodium, serum

CBC, which includes:

- Hematocrit
- Hemoglobin
- MCV
- MCH
- MCHC
- RDW
- Percentage and absolute differential counts
- RBC
- WBC

Urinalysis, which includes:

- Color
- Appearance
- Specific gravity
- pH
- Protein
- Glucose
- Ketones
- Occult blood
- Leukocyte esterase
- Nitrite
- Bilirubin
- Urobilinogen

Participants who test positive on the qualitative urine drug test at both pre and post cessation timepoints will have a Quantitative Urine THC-COOH Drug Test to determine likelihood of compliance with abstinence requirements. Quantitative testing will be conducted by Dominion Diagnostics using GC/MS testing methods. An algorithm developed by Huestis and colleagues will then be used to determine the confidence that a participant completely abstained from cannabis during each 2-week cessation period. Participants for which samples indicate 90% or greater confidence will be considered compliant. Participants who provide samples that indicate 80-90% confidence will have blood samples tested for additional information in order to determine compliance. Samples with < 80% confidence will be considered non-compliant. Non-compliance will

be evaluated and handled by study investigators on a case-by-case basis with regards to study continuation and utilization of data.

Thyroid function, which includes:

- TSH high sensitivity

HIV serology will be performed.

A urine-dip pregnancy test for participants of childbearing potential will be performed as specified in the Time & Events table.

A qualitative urine drug screen will be administered at Screen 1 and 2. A qualitative urine drug screen will be administered throughout the study at the study site according to the Time & Events table.

An 80-hour ETG urine dip test to test for recent alcohol consumption will be performed at the study site according to the Time & Events table.

Clinical laboratory assessments will be drawn at the site or closest available LabCorp facility. These samples will be processed at LabCorp.

9465 E Ironwood Square Dr. 103
Scottsdale, AZ 85258

10.2 Blood Cannabinoid Analysis

For participants who test positive for THC-COOH (50ng/mL cut-off) on the qualitative urine drug tests during screening and who also show inconclusive cessation results from quantitative urine analysis, an accredited and certified laboratory will conduct testing of blood cannabinoids (THC, 11-OH-THC, and THC-COOH) using high performance liquid chromatography-tandem mass spectrometry (LC-MS/MS).

10.3 Research Lab Biomarker Analysis

Biomarker laboratory analyses will be performed using plasma for CRP, IL-1β, and IL-6 levels collected from all participants according to the Time and Events Table. For blood samples, a butterfly needle will be used to draw 30mL of blood into chilled EDTA vacutainer tubes. Samples will be immediately placed on wet ice. Plasma will be extracted by centrifugation in a refrigerated (4 °C) centrifuge at 1,300g for 10 minutes, aliquotted into individual tubes, and stored at -80 °C prior to shipment to the collaborating site at University of Colorado Boulder. Biomarker analyses will be performed by David Smith in Dr. Lowry's laboratory at the University of Colorado Boulder in Boulder, Colorado (collaborating site). Concentrations of CRP, IL-1β, and IL-6 will be quantified using commercially available enzyme immunoassay kits (R&D Systems, Minneapolis, MN, USA). These kits are highly specific and exclusively

recognize CRP, IL-1β, and IL-6 respectively, and not related peptides. Plasma samples are assayed in duplicate and measured using a microplate reader for 96-well format according to manufacturer's instructions.

11.0 Study Monitoring, Auditing and Documentation

Clinical Investigators and/or their site staff will be trained prior to the start of the protocol by the sponsor's clinical research staff. The clinical study site will be monitored by site visits and remote communication to the CI by representatives of the sponsor. The site will be monitored as appropriate for the rate of enrollment. During each monitoring visit, source data verification will be performed by a Clinical Research Associate (CRA) to ensure compliance, including accurate and complete recording of data on CRFs, source documents, and drug accountability records. CRFs supplied by the sponsor will be completed for each participant enrolled. Monitoring and auditing procedures of the sponsor will be followed, in order to comply with Good Clinical Practice (GCP) guidelines and to ensure validity of the study data.

The sponsor will review the study documentation used for planning, conducting and monitoring of the study in order to ensure compliance with GCP and local regulations. This documentation includes, at minimum: the Investigator's Brochure, the Study Protocol, the CRFs and the ICF.

During or after the clinical protocol, the regulatory authorities, the IRB, and/or representatives of the sponsor may request access to all source documents, CRFs and other protocol documentation for on-site audit or inspection.

12.0 Data Analysis

Key Personnel, MAPS, and the biostatistician will agree on a detailed Statistical Analysis Plan prior to the beginning of recruitment. The biostatistician will perform a "modified intent to treat" (mITT) analysis using repeated measures analysis of variance (ANOVA) to compare demographics and baseline characteristics. Distribution of treatment dropouts will be examined by condition and if found to be unequal, the sponsor will control for group assignment in the mITT analysis.

Primary analysis of observer-blind CAPS-5 data collected at Baseline and end of Stage 1 will be conducted using ANCOVA with marijuana concentration as a between-subject factor and time of assessment as a within-group factor. If effects of marijuana concentration are detected at Stage 1, a similar secondary analysis will also be performed with Stage 2 data using Stage 1 data as a covariate for each outcome measure. Effect size will be estimated based on all outcome measures using Cohen's techniques. The biostatistician will conduct secondary exploratory analyses of weekly assessments of PCL-5, ISI, IDAS, IPF, C-SSRS, MWC, CUDIT-R and ACT scores using repeated measures ANCOVA or generalized linear mixed modeling, as described in the Statistical Analysis Plan. Statistical significance ($p < .05$) will be determined for all tests. All available data from repeated outcome assessments of all participants who are

randomized, even if they withdraw from the allocated treatment prior to the primary endpoints, will be included in mITT analysis.

A repeated measures ANOVA comparison of introductory session ratings on the DEQ and pulse will be conducted, with marijuana condition as the main factor in the analysis. If a main effect of marijuana concentration is observed then pairwise comparisons will be conducted to detect differences between each marijuana concentration.

Secondary exploratory analyses will be conducted examining weekly assessments of self-reported (via the ISI) and objective (via Actigraphy) measures of sleep. Independent repeated measures ANOVAs will be conducted. In terms of self-reported sleep, weekly total scores from the ISI will be entered as the repeated measure with group entered as the IV. In terms of objective sleep (via Actigraphy) repeated measures ANCOVAs will be conducted with weekly total sleep time (TST), wake after sleep onset (WASO), sleep onset latency (SOL), and sleep efficiency (SE) entered as the repeated measure in independent analyses and group entered as the IV. Statistical significance will be set at p=.05 for all analyses.

Quantitative levels of THC, CBD and their metabolites will be assessed in order to ascertain whether participants comply with only using the marijuana assigned to them during the study. If there is sufficient data, correlational analyses may also be conducted between biological cannabinoid levels and clinical outcomes to determine whether there is a dose-response effect between cannabinoid exposure and clinical response.

Prognostic value of CRP, IL-1β and Il-6 will be examined using two regression analyses, one between biomarker values gathered at Baseline and at the primary endpoint CAPS-5 global severity score, and another between biomarker values collected after two weeks of cessation and the CAPS-5 global severity score at the end of Stage 1.

Qualitative safety analyses will examine Daily Diaries with summary tables listing maximum severity and duration, concomitant medications/therapies, and AEs with frequencies and percentages tabulated overall and by concentration group. Spontaneously reported reactions will be separated from unexpected AEs during analysis and presented separately in summary tables. Statistical analyses will be conducted to evaluate safety and AEs by marijuana treatment condition and individual demographic characteristics (e.g. gender, history of cannabis use, concomitant medication/drug use, baseline CAPS-5 score).

12.1 Statistical Power

The proposed study is a pilot investigation intended to gather preliminary data on the safety and efficacy of marijuana in people with PTSD. Because of their exploratory nature, pilot studies are often not powered for detecting the desired effect. Because it is a pilot study in a small sample, results will used to assess variability of the outcome

measures as inputs for calculation of sample sizes for adequately powered subsequent studies. This study will be the first to examine the effects of marijuana on PTSD.

This pilot RCT is the first study of its kind intended to gather estimates of effect size of marijuana for PTSD. In the absence of published effect sizes of marijuana for PTSD, possible effect sizes are estimated to be 0.4 (small to medium effect) for a between-subject comparison to ensure an adequately powered study size, based on a recent meta-analysis conducted with the National Center for PTSD [170]. Thus in the proposed study, N=76 with 19 participants randomized to each group will have 82% power to detect these or greater differences between groups on the same primary outcome measure (change in CAPS-5 total score) used to obtain FDA approval for PTSD medications. Similar calculations would apply for secondary analyses.

13.0 Informed Consent

The CI or co-investigators are responsible for obtaining informed consent in adherence to GCP and according to applicable regulations prior to entering the participant into the trial. Information about events during the course of the study must be given both orally and written, in an understandable form. In addition to the explanation of evaluation, introductory sessions, periods of marijuana self-administration, and periods of abstinence from marijuana, the information should include that access to original medical records and processing of coded personal information must be authorized. The informed consent discussion must be conducted by a person who is qualified, according to applicable local regulations and GCP. The participant should have the opportunity to inquire about details of the study and to consider whether or not to participate.

The ICF must be signed and dated by the participant and must be countersigned by the site staff administering the consent process.

Site staff will provide a copy of the signed ICF to the participant, and will maintain the original in the investigator’s study file. During the study, participants will be reminded of upcoming study procedures at each visit to assure continued comprehension of the ICF.

The written ICF and any other written information to be provided to participants should be revised whenever important new information becomes available that may be relevant to the participant's consent. Any revised written informed consent form, and written information, should receive approval from an IRB before use. Written consent to take part in the study session includes giving the CI permission to view the participant's recent medical records to assess study eligibility, if needed. Information necessary for study participation includes past medical history, psychiatric interview, physical examination, and clinical laboratory tests.

The participant should be informed in a timely manner if new information becomes available that may affect the decision to take part in the study. The communication of this information should be documented.

Participants can withdraw consent for participation in the protocol at any time without prejudice. If a participant withdraws consent but does not revoke the Health Insurance Portability and Accountability Act (HIPAA) authorization or equivalent form, MAPS will have full access to the participant's medical records, including termination visit information. If a participant revokes only the HIPAA authorization, MAPS will have full access to all of the participant's medical records prior to the date and time of revocation.

13.1 Confidentiality

Every effort will be made to strictly safeguard the confidentiality of participants in their role as research participants. Participant Contact Information logs will be kept on paper at the clinical trial sites in locked secure files. Removing identifying information from data and restricting access to researchers directly involved in assessing the participants should prevent the dissemination of confidential data, with or without identifying information. Despite this, privacy cannot be guaranteed. Except for the screening log, the ICF, and a participant contact information sheet, all data will be identified only by the participant's initials on the source document and five-digit participant number numeric code. If past medical records are needed, participants will sign forms for the release of information upon consent to permit screening for protocol enrollment. All assessment records will be kept in a locked file drawer or cabinet, and access to measures will be limited to regulatory agencies, researchers, and individuals analyzing data. Researchers, other than the CI directly involved in the protocol, with access to data will not be provided with any information that would identify participants by name or by other means, such as social security number. Staff viewing participant video recordings of marijuana use will sign a confidentiality form. Files will be uploaded on a secure computer in a locked office, and only the date of the recording and participant number will be retained in these files.

Maintaining data in a secure environment will prevent the accidental or deliberate examination or removal of data. The sponsor will utilize confidentiality procedures to assure participant privacy. Data to be transferred to remote servers will be encrypted during transfer using a Virtual Private Network. Clinical trial data other than video data will be hosted on Medrio, an Electronic Data Capture (EDC) system that is FDA-compliant. All data entered into this system will be de-identified. Participants will only be referred to by numbers and a secondary identifier code. Source Records and identifying information will be retained at the clinical site per GCP. The sponsor will train the study staff on EDC procedures. Each study staff member with access to the data will be given an individual password.

The sponsor has developed a feature that will allow participants to create a password and enter their self-report questionnaire data directly into Medrio using the ePRO feature. Participants will be reminded by email to enter the data. Participant emails will be treated as Protected Health Information (PHI) in the database. Participants will receive a welcome email and reminder emails to ensure that they provide all necessary data.

Subjects will be showed how to securely use their tablet computers. This will include instruction on password protection, ePRO and video uploads to ensure security. After the upload is confirmed as complete at the site, the original video on the handheld device will be deleted by site staff. All identifying information and study related information will be stored in locked files, encrypted drives, or secure servers. If a tablet is lost or stolen, local police will be notified and every effort made to recover the device. Participants will be instructed to store their tablet computers in their study supplied lock box.

13.2 Costs to Participants and Compensation

The Sponsor of this study will cover the costs that are directly related to this study. This includes the costs for introductory sessions, for the psychological and laboratory testing, for medical examinations, the portable tablet with video and lockable storage box, and the study drug. Neither the participant nor their medical insurance (if any) will be charged for any procedures done solely for the purpose of the study. The subject will not be charged for lost or damaged tablet computers.

Charges for treatment of the participant's condition that are unrelated to the research study, or any of its procedures, will continue to be billed to the health insurance provider of the participant or to the participant him or herself. It is anticipated that there will not be any charges for treatment that is unrelated to the study.

Participants will be compensated for their time and effort, up to $1100 for completion of Stage 1 and Stage 2. Compensation will be pro-rated for visits and tasks as they are completed. Compensation of this magnitude is appropriate given the length and nature of this study.

Table 7: Compensation by Study Stage

Study Stage	Compensation Amount	Total Compensation
Screen Failure	$20/week	$40 total
Stage 1	$125/week	$375 total
Cessation 1 (Completion Bonus)	$75/week	$150 total
Stage 2	$125/week	$375 total
Cessation 2 (Plus Completion Bonus)	$100/week	$200 total
Stage 3 (optional)	No Compensation	$0 total
Total Possible Compensation	---	$1100 total

13.3 Treatment and Compensation for Study Related Injury

Treatment of a study-related emergency would first be billed to a participant's health insurance provider. The sponsor will cover any direct costs relating to the treatment of a study-related emergency that are not covered by a participant's health insurance. The study involves self-administration of a study drug. Participants will be directed to go to the nearest emergency department if they experience a medical emergency, and the sponsor will provide compensation for study-related medical treatment costs.

14.0 Record Retention

CI must retain all study records required by MAPS and by the applicable regulations in a secure and safe facility. The CI must consult a MAPS representative before disposal of any study records. “Essential documents” are defined as documents that individually and collectively permit evaluation of the conduct of a trial and the quality of the data produced. It is the responsibility of the sponsor to inform the CI/institution as to when these documents no longer need to be retained. CI must retain all study records required by MAPS and by the applicable regulations in a secure and safe facility. The CI must consult a MAPS representative before disposal of any study records.

15.0 Publication Policy

The sponsor recognizes the importance of communicating medical study data and therefore encourages publications in reputable scientific journals and presentations at seminars or conferences. It is understood by the investigators that the information generated in this study will be used by the sponsor in connection with the development of the product and therefore may be disclosed to government agencies in various countries. To allow for the use of information derived from the study, it is understood that the CI is obliged to provide the sponsor with complete test results, all study data, and access to all study records.

Any results of medical investigations with the sponsor products and/or publication/lecture/manuscripts based thereon, shall be exchanged and discussed by the investigators and the sponsor clinical research representative(s) prior to submission for publication or presentation. Due regard shall be given to the sponsor's legitimate interests, e.g. manuscript authorship, obtaining optimal patient protection, coordinating and maintaining submissions to health authorities, and coordinating with other ongoing studies in the same field.

The full details of the processes of producing and reviewing reports, manuscripts, and presentations based on the data from this trial will be described in the Clinical Trial Agreement.

16.0 References

1. Mithoefer, M.C., et al., *The safety and efficacy of {+/-}3,4-methylenedioxymethamphetamine-assisted psychotherapy in subjects with chronic, treatment-resistant posttraumatic stress disorder: the first randomized controlled pilot study.* J Psychopharmacol, 2011. **25**(4): p. 439-52.
2. Mithoefer, M.C., et al., *Durability of improvement in post-traumatic stress disorder symptoms and absence of harmful effects or drug dependency after 3,4-methylenedioxymethamphetamine-assisted psychotherapy: a prospective long-term follow-up study.* J Psychopharmacol, 2013. **27**(1): p. 28-39.
3. Oehen, P., et al., *A randomized, controlled pilot study of MDMA (+/- 3,4-Methylenedioxymethamphetamine)-assisted psychotherapy for treatment of resistant, chronic Post-Traumatic Stress Disorder (PTSD).* J Psychopharmacol, 2013. **27**(1): p. 40-52.
4. Bonn-Miller, M.O., K.A. Babson, and R. Vandrey, *Using cannabis to help you sleep: heightened frequency of medical cannabis use among those with PTSD.* Drug Alcohol Depend, 2014. **136**: p. 162-5.
5. Procon.org. *23 Legal Medical Marijuana States and DC; Medical Marijuana - Procon.org*. 2014 [cited 2014 December 9, 2014]; Available from: http://medicalmarijuana.procon.org/view.resource.php?resourceID=000881.
6. *Medical Cannabis Numbers as of August 18, 2010.* 2010, New Mexico Department of Health Medical Cannabis Program: http://nmhealth.org/IDB/medicalcannabis/Medical%20Cannabis%20Numbers%20as%20of%208-18-10.pdf.
7. Bonn-Miller, M.O., et al., *Self-reported cannabis use characteristics, patterns and helpfulness among medical cannabis users.* Am J Drug Alcohol Abuse, 2014. **40**(1): p. 23-30.
8. Bonn-Miller, M.O., A.H. Harris, and J.A. Trafton, *Prevalence of cannabis use disorder diagnoses among veterans in 2002, 2008, and 2009.* Psychol Serv, 2012. **9**(4): p. 404-16.
9. Kessler, R.C., et al., *Lifetime prevalence and age-of-onset distributions of DSM-IV disorders in the National Comorbidity Survey Replication.* Arch Gen Psychiatry, 2005. **62**(6): p. 593-602.
10. Resnick, H.S., et al., *Prevalence of civilian trauma and posttraumatic stress disorder in a representative national sample of women.* J Consult Clin Psychol, 1993. **61**(6): p. 984-91.
11. Brunello, N., et al., *Posttraumatic stress disorder: diagnosis and epidemiology, comorbidity and social consequences, biology and treatment.* Neuropsychobiology, 2001. **43**(3): p. 150-62.
12. Norris, F.H., et al., *Epidemiology of trauma and posttraumatic stress disorder in Mexico.* J Abnorm Psychol, 2003. **112**(4): p. 646-56.
13. Perkonigg, A., et al., *Traumatic events and post-traumatic stress disorder in the community: prevalence, risk factors and comorbidity.* Acta Psychiatr Scand, 2000. **101**(1): p. 46-59.

14. Sareen, J., et al., *Disability and poor quality of life associated with comorbid anxiety disorders and physical conditions.* Arch Intern Med, 2006. **166**(19): p. 2109-16.
15. Zlotnick, C., et al., *Epidemiology of trauma, post-traumatic stress disorder (PTSD) and co-morbid disorders in Chile.* Psychol Med, 2006. **36**(11): p. 1523-33.
16. Hoge, C.W., et al., *Combat duty in Iraq and Afghanistan, mental health problems, and barriers to care.* N Engl J Med, 2004. **351**(1): p. 13-22.
17. Tanielian, T.L., L. Jaycox, and Rand Corporation., *Invisible wounds of war : psychological and cognitive injuries, their consequences, and services to assist recovery*. 2008, Santa Monica, CA: RAND. xliii, 453 p.
18. *Statement Of Jon A. Wooditch Acting Inspector General Department Of Veterans Affairs*, in *Committee On Veterans' Affairs Subcommittee On Disability Assistance And Memorial Affairs*. 2005: Washington, DC.
19. de Jong, J.T., I.H. Komproe, and M. Van Ommeren, *Common mental disorders in postconflict settings.* Lancet, 2003. **361**(9375): p. 2128-30.
20. Thabet, A.A. and P. Vostanis, *Post-traumatic stress reactions in children of war.* J Child Psychol Psychiatry, 1999. **40**(3): p. 385-91.
21. Weine, S.M., et al., *Psychiatric consequences of "ethnic cleansing": clinical assessments and trauma testimonies of newly resettled Bosnian refugees.* Am J Psychiatry, 1995. **152**(4): p. 536-42.
22. Kessler, R.C., et al., *Prevalence, severity, and comorbidity of 12-month DSM-IV disorders in the National Comorbidity Survey Replication.* Arch Gen Psychiatry, 2005. **62**(6): p. 617-27.
23. Breslau, N. and G.C. Davis, *Posttraumatic stress disorder in an urban population of young adults: risk factors for chronicity.* Am J Psychiatry, 1992. **149**(5): p. 671-5.
24. Breslau, N., *The epidemiology of posttraumatic stress disorder: what is the extent of the problem?* J Clin Psychiatry, 2001. **62 Suppl 17**: p. 16-22.
25. Frayne, S.M., et al., *Burden of medical illness in women with depression and posttraumatic stress disorder.* Arch Intern Med, 2004. **164**(12): p. 1306-12.
26. Foa, E.B., et al., *Effective Treatments for PTSD, Practice Guidelines from the International Society for Traumatic Stress Studies*. Second ed. 2009, New York, NY: Guilford Press.
27. Benedek, D.M., et al. *Guideline watch (March 2009): Practice guideline for the treatment of patients with acute stress disorder and posttraumatic stress disorder*. Psychiatry Online, 2009.
28. Foa, E.B., et al., *A comparison of exposure therapy, stress inoculation training, and their combination for reducing posttraumatic stress disorder in female assault victims.* J Consult Clin Psychol, 1999. **67**(2): p. 194-200.
29. Resick, P.A. and M.K. Schnicke, *Cognitive processing therapy for sexual assault victims.* J Consult Clin Psychol, 1992. **60**(5): p. 748-56.
30. van der Kolk, B.A., et al., *Fluoxetine in posttraumatic stress disorder.* J Clin Psychiatry, 1994. **55**(12): p. 517-22.
31. Brady, K., et al., *Efficacy and safety of sertraline treatment of posttraumatic stress disorder: a randomized controlled trial.* JAMA, 2000. **283**(14): p. 1837-44.

32. American Psychiatric, A., *Diagnostic and Statistical Manual of Mental Disorders (DSM IV): 4th Edition*. 4th ed. 1994, Washington, DC: American Psychiatric Publishing.
33. Jordan, R.G., T.V. Nunley, and R.R. COOK, *Symptom exaggeration in a PTSD inpatient population: Response set or claim for compensation.* J Trauma Stress, 1992. **5**: p. 633-642.
34. Smith, D.W. and B.C. Frueh, *Compensation seeking, comorbidity, and apparent exaggeration of PTSD symptoms among Vietnam combat veterans. .* Psychological Assessment, 1996. **8**: p. 3-6.
35. Calhoun, P.S., et al., *Drug use and validity of substance use self-reports in veterans seeking help for posttraumatic stress disorder.* J Consult Clin Psychol, 2000. **68**(5): p. 923-7.
36. Schlicker, E. and M. Kathmann, *Modulation of transmitter release via presynaptic cannabinoid receptors.* Trends Pharmacol Sci, 2001. **22**(11): p. 565-72.
37. Greer, G.R., C.S. Grob, and A.L. Halberstadt, *PTSD symptom reports of patients evaluated for the New Mexico Medical Cannabis Program.* J Psychoactive Drugs, 2014. **46**(1): p. 73-7.
38. Passie, T., et al., *Mitigation of post-traumatic stress symptoms by Cannabis resin: a review of the clinical and neurobiological evidence.* Drug Test Anal, 2012. **4**(7-8): p. 649-59.
39. Roitman, P., et al., *Preliminary, open-label, pilot study of add-on oral Delta9-tetrahydrocannabinol in chronic post-traumatic stress disorder.* Clin Drug Investig, 2014. **34**(8): p. 587-91.
40. Cougle, J.R., et al., *Posttraumatic stress disorder and cannabis use in a nationally representative sample.* Psychol Addict Behav, 2011. **25**(3): p. 554-8.
41. Chhatwal, J.P. and K.J. Ressler, *Modulation of fear and anxiety by the endogenous cannabinoid system.* CNS Spectr, 2007. **12**(3): p. 211-20.
42. Pistis, M., et al., *Cannabinoids modulate neuronal firing in the rat basolateral amygdala: evidence for CB1- and non-CB1-mediated actions.* Neuropharmacology, 2004. **46**(1): p. 115-25.
43. Hajos, N. and T.F. Freund, *Pharmacological separation of cannabinoid sensitive receptors on hippocampal excitatory and inhibitory fibers.* Neuropharmacology, 2002. **43**(4): p. 503-10.
44. Neumeister, A., et al., *Elevated brain cannabinoid CB1 receptor availability in post-traumatic stress disorder: a positron emission tomography study.* Mol Psychiatry, 2013. **18**(9): p. 1034-40.
45. Patel, S. and C.J. Hillard, *Adaptations in endocannabinoid signaling in response to repeated homotypic stress: a novel mechanism for stress habituation.* Eur J Neurosci, 2008. **27**(11): p. 2821-9.
46. Arevalo, C., R. de Miguel, and R. Hernandez-Tristan, *Cannabinoid effects on anxiety-related behaviours and hypothalamic neurotransmitters.* Pharmacol Biochem Behav, 2001. **70**(1): p. 123-31.
47. Navarro, M., et al., *Acute administration of the CB1 cannabinoid receptor antagonist SR 141716A induces anxiety-like responses in the rat.* Neuroreport, 1997. **8**(2): p. 491-6.

48. Beinfeld, M.C. and K. Connolly, *Activation of CB1 cannabinoid receptors in rat hippocampal slices inhibits potassium-evoked cholecystokinin release, a possible mechanism contributing to the spatial memory defects produced by cannabinoids.* Neurosci Lett, 2001. **301**(1): p. 69-71.
49. Katona, I., et al., *Presynaptically located CB1 cannabinoid receptors regulate GABA release from axon terminals of specific hippocampal interneurons.* J Neurosci, 1999. **19**(11): p. 4544-58.
50. Marsicano, G. and B. Lutz, *Expression of the cannabinoid receptor CB1 in distinct neuronal subpopulations in the adult mouse forebrain.* Eur J Neurosci, 1999. **11**(12): p. 4213-25.
51. Onaivi, E.S., M.R. Green, and B.R. Martin, *Pharmacological characterization of cannabinoids in the elevated plus maze.* J Pharmacol Exp Ther, 1990. **253**(3): p. 1002-9.
52. Houser, S.J., et al., *Dynorphin B and spinal analgesia: induction of antinociception by the cannabinoids CP55,940, Delta(9)-THC and anandamide.* Brain Res, 2000. **857**(1-2): p. 337-42.
53. Pugh, G., Jr., et al., *Involvement of dynorphin B in the antinociceptive effects of the cannabinoid CP55,940 in the spinal cord.* J Pharmacol Exp Ther, 1997. **281**(2): p. 730-7.
54. Zimmer, A., et al., *Absence of delta -9-tetrahydrocannabinol dysphoric effects in dynorphin-deficient mice.* J Neurosci, 2001. **21**(23): p. 9499-505.
55. Berrendero, F. and R. Maldonado, *Involvement of the opioid system in the anxiolytic-like effects induced by Delta(9)-tetrahydrocannabinol.* Psychopharmacology (Berl), 2002. **163**(1): p. 111-7.
56. Marin, S., et al., *Involvement of the kappa-opioid receptor in the anxiogenic-like effect of CP 55,940 in male rats.* Pharmacol Biochem Behav, 2003. **74**(3): p. 649-56.
57. Marsicano, G., et al., *The endogenous cannabinoid system controls extinction of aversive memories.* Nature, 2002. **418**(6897): p. 530-4.
58. Kuhnert, S., C. Meyer, and M. Koch, *Involvement of cannabinoid receptors in the amygdala and prefrontal cortex of rats in fear learning, consolidation, retrieval and extinction.* Behav Brain Res, 2013. **250**: p. 274-84.
59. Ganon-Elazar, E. and I. Akirav, *Cannabinoids and traumatic stress modulation of contextual fear extinction and GR expression in the amygdala-hippocampal-prefrontal circuit.* Psychoneuroendocrinology, 2013. **38**(9): p. 1675-87.
60. Fraser, G.A., *The use of a synthetic cannabinoid in the management of treatment-resistant nightmares in posttraumatic stress disorder (PTSD).* CNS Neurosci Ther, 2009. **15**(1): p. 84-8.
61. Berger, W., et al., *Pharmacologic alternatives to antidepressants in posttraumatic stress disorder: a systematic review.* Prog Neuropsychopharmacol Biol Psychiatry, 2009. **33**(2): p. 169-80.
62. Zanelati, T.V., et al., *Antidepressant-like effects of cannabidiol in mice: possible involvement of 5-HT1A receptors.* Br J Pharmacol, 2010. **159**(1): p. 122-8.
63. Cameron, C., D. Watson, and J. Robinson, *Use of a synthetic cannabinoid in a correctional population for posttraumatic stress disorder-related insomnia and*

nightmares, chronic pain, harm reduction, and other indications: a retrospective evaluation. J Clin Psychopharmacol, 2014. **34**(5): p. 559-64.

64. Jetly, R., et al., *The efficacy of nabilone, a synthetic cannabinoid, in the treatment of PTSD-associated nightmares: A preliminary randomized, double-blind, placebo-controlled cross-over design study.* Psychoneuroendocrinology, 2014.
65. Bhattacharyya, S., et al., *Opposite effects of delta-9-tetrahydrocannabinol and cannabidiol on human brain function and psychopathology.* Neuropsychopharmacology, 2010. **35**(3): p. 764-74.
66. Crippa, J.A., et al., *Cannabis and anxiety: a critical review of the evidence.* Hum Psychopharmacol, 2009. **24**(7): p. 515-23.
67. Morgan, C.J., et al., *Impact of cannabidiol on the acute memory and psychotomimetic effects of smoked cannabis: naturalistic study.* Br J Psychiatry, 2010. **197**: p. 285-90.
68. Fusar-Poli, P., et al., *Distinct effects of {delta}9-tetrahydrocannabinol and cannabidiol on neural activation during emotional processing.* Arch Gen Psychiatry, 2009. **66**(1): p. 95-105.
69. Rauch, S.L., et al., *Exaggerated amygdala response to masked facial stimuli in posttraumatic stress disorder: a functional MRI study.* Biol Psychiatry, 2000. **47**(9): p. 769-76.
70. Eraly, S.A., et al., *Assessment of plasma C-reactive protein as a biomarker of posttraumatic stress disorder risk.* JAMA Psychiatry, 2014. **71**(4): p. 423-31.
71. Tucker, P., et al., *Neuroimmune and cortisol changes in selective serotonin reuptake inhibitor and placebo treatment of chronic posttraumatic stress disorder.* Biol Psychiatry, 2004. **56**(2): p. 121-8.
72. Newton, T.L., et al., *Interleukin-6 and soluble interleukin-6 receptor levels in posttraumatic stress disorder: associations with lifetime diagnostic status and psychological context.* Biol Psychol, 2014. **99**: p. 150-9.
73. Tursich, M., et al., *Association of trauma exposure with proinflammatory activity: a transdiagnostic meta-analysis.* Transl Psychiatry, 2014. **4**: p. e413.
74. Gill, J.M., et al., *Women in recovery from PTSD have similar inflammation and quality of life as non-traumatized controls.* J Psychosom Res, 2013. **74**(4): p. 301-6.
75. Mechoulam, R., et al., *Cannabidiol--recent advances.* Chem Biodivers, 2007. **4**(8): p. 1678-92.
76. Rajavashisth, T.B., et al., *Decreased prevalence of diabetes in marijuana users: cross-sectional data from the National Health and Nutrition Examination Survey (NHANES) III.* BMJ Open, 2012. **2**: p. e000494.
77. Huestis, M.A., A. Barnes, and M.L. Smith, *Estimating the time of last cannabis use from plasma delta9-tetrahydrocannabinol and 11-nor-9-carboxy-delta9-tetrahydrocannabinol concentrations.* Clin Chem, 2005. **51**(12): p. 2289-95.
78. Weathers, F.W., et al., *The Clinician-Administered PTSD Scale for DSM-5 (CAPS-5).* 2013, Washington DC: National Center for PTSD.
79. American Psychiatric Association, *Diagnostic and statistical manual of mental disorders : DSM-5.* 5th ed. 2013, Washington, D.C.: American Psychiatric Association. xliv, 947 p.

80. Weathers, F.W., et al., *The PTSD Checklist for DSM-5 (PCL-5).* National Center for PTSD, 2013.
81. Watson, D., et al., *Development and validation of the Inventory of Depression and Anxiety Symptoms (IDAS).* Psychol Assess, 2007. **19**(3): p. 253-68.
82. Watson, D., et al., *Further validation of the IDAS: evidence of convergent, discriminant, criterion, and incremental validity.* Psychol Assess, 2008. **20**(3): p. 248-59.
83. Morin, C.M., *Insomnia: psychological assessment and management*. 1993, New York, NY: Guilford Press.
84. Bastien, C.H., A. Vallieres, and C.M. Morin, *Validation of the Insomnia Severity Index as an outcome measure for insomnia research.* Sleep Med, 2001. **2**(4): p. 297-307.
85. Morin, C.M., et al., *The Insomnia Severity Index: psychometric indicators to detect insomnia cases and evaluate treatment response.* Sleep, 2011. **34**(5): p. 601-8.
86. Yang, M., et al., *Interpreting score differences in the Insomnia Severity Index: using health-related outcomes to define the minimally important difference.* Curr Med Res Opin, 2009. **25**(10): p. 2487-94.
87. Marx, B.P. and E. al., *Development of a functional evaluation scale for active duty service members and veterans*, in *International Society for Traumatic Stress Studies*. 2009: Atlanta, GA.
88. Sobell, M.B., et al., *The reliability of a timeline method for assessing normal drinker college students' recent drinking history: utility for alcohol research.* Addict Behav, 1986. **11**(2): p. 149-61.
89. Mariani, J.J., et al., *Quantification and comparison of marijuana smoking practices: blunts, joints, and pipes.* Drug Alcohol Depend, 2011. **113**(2-3): p. 249-51.
90. Adamson, S.J., et al., *An improved brief measure of cannabis misuse: the Cannabis Use Disorders Identification Test-Revised (CUDIT-R).* Drug Alcohol Depend, 2010. **110**(1-2): p. 137-43.
91. Budney, A.J., P.L. Novy, and J.R. Hughes, *Marijuana withdrawal among adults seeking treatment for marijuana dependence.* Addiction, 1999. **94**(9): p. 1311-22.
92. Budney, A.J., et al., *The time course and significance of cannabis withdrawal.* J Abnorm Psychol, 2003. **112**(3): p. 393-402.
93. Budney, A.J., et al., *Comparison of cannabis and tobacco withdrawal: severity and contribution to relapse.* J Subst Abuse Treat, 2008. **35**(4): p. 362-8.
94. Vandrey, R., et al., *The dose effects of short-term dronabinol (oral THC) maintenance in daily cannabis users.* Drug Alcohol Depend, 2013. **128**(1-2): p. 64-70.
95. Vandrey, R., A. Umbricht, and E.C. Strain, *Increased blood pressure after abrupt cessation of daily cannabis use.* J Addict Med, 2011. **5**(1): p. 16-20.
96. Vandrey, R.G., et al., *A within-subject comparison of withdrawal symptoms during abstinence from cannabis, tobacco, and both substances.* Drug Alcohol Depend, 2008. **92**(1-3): p. 48-54.

97. Posner, K., et al., *Columbia Classification Algorithm of Suicide Assessment (C-CASA): classification of suicidal events in the FDA's pediatric suicidal risk analysis of antidepressants.* Am J Psychiatry, 2007. **164**(7): p. 1035-43.
98. Morean, M.E., et al., *The drug effects questionnaire: psychometric support across three drug types.* Psychopharmacology (Berl), 2013. **227**(1): p. 177-92.
99. Weafer, J. and H. de Wit, *Inattention, impulsive action, and subjective response to D-amphetamine.* Drug Alcohol Depend, 2013. **133**(1): p. 127-33.
100. Kirkpatrick, M.G., et al., *MDMA effects consistent across laboratories.* Psychopharmacology (Berl), 2014.
101. First MB, et al., *Structured Clinical Interview for DSM-5 - Research Version (SCID-5 for DSM-5, Research Version; (SCID-5-RV)*. 2015, Arlington, VA: American Psychiatric Association.
102. Ross, C.A., et al., *Differentiating Multiple Personality Disorder and Dissociative Disorder Not Otherwise Specified.* Dissociation, 1992. **5**(2): p. 87-90.
103. Clark, R.C. and D.P. Watson, *Cannabis and Natural Cannabis Medicines*, in *Forensics and Science: Marijuana and the Cannabinoids*, M.A. ElSohly, Editor. 2007, Humana Press: Totowa, NJ. p. 1-15.
104. Grinspoon, L., *Medical marihuana in a time of prohibition.* Int J Drug Policy, 1999. **10** (2): p. 145-156.
105. Ilan, A.B., et al., *Neurophysiological and subjective profile of marijuana with varying concentrations of cannabinoids.* Behav Pharmacol, 2005. **16**(5-6): p. 487-96.
106. Russo, E. and G.W. Guy, *A tale of two cannabinoids: the therapeutic rationale for combining tetrahydrocannabinol and cannabidiol.* Med Hypotheses, 2006. **66**(2): p. 234-46.
107. Zuardi, A.W., et al., *Cannabidiol, a Cannabis sativa constituent, as an antipsychotic drug.* Braz J Med Biol Res, 2006. **39**(4): p. 421-9.
108. Khalsa, J.H., *Medical and Health Consequences of Marijuana*, in *Forensic Science and Medicine: Marijuana and the Cannabinoids*, M.A. ElSohly, Editor. 2007, Humana Publishing: Totowa, NJ. p. 237-252.
109. Zuurman, L., et al., *Biomarkers for the effects of cannabis and THC in healthy volunteers.* Br J Clin Pharmacol, 2009. **67**(1): p. 5-21.
110. Battistella, G., et al., *Weed or wheel! FMRI, behavioural, and toxicological investigations of how cannabis smoking affects skills necessary for driving.* PLoS One, 2013. **8**(1): p. e52545.
111. Downey, L.A., et al., *The effects of cannabis and alcohol on simulated driving: Influences of dose and experience.* Accid Anal Prev, 2013. **50**: p. 879-86.
112. Downey, L.A., et al., *Detecting impairment associated with cannabis with and without alcohol on the Standardized Field Sobriety Tests.* Psychopharmacology (Berl), 2012. **224**(4): p. 581-9.
113. Sewell, R.A., M. Ranganathan, and D.C. D'Souza, *Cannabinoids and psychosis.* Int Rev Psychiatry, 2009. **21**(2): p. 152-62.
114. Degenhardt, L. and W. Hall, *Is cannabis use a contributory cause of psychosis?* Can J Psychiatry, 2006. **51**(9): p. 556-65.
115. Kouri, E., et al., *Attributes of heavy vs. occasional marijuana smokers in a college population.* Biol Psychiatry, 1995. **38**(7): p. 475-81.

116. Pope, H.G., Jr. and D. Yurgelun-Todd, *The residual cognitive effects of heavy marijuana use in college students.* Jama, 1996. **275**(7): p. 521-7.
117. Solowij, N., et al., *Biopsychosocial changes associated with cessation of cannabis use: a single case study of acute and chronic cognitive effects, withdrawal and treatment.* Life Sci, 1995. **56**(23-24): p. 2127-34.
118. Bosker, W.M., et al., *Psychomotor function in chronic daily Cannabis smokers during sustained abstinence.* PLoS One, 2013. **8**(1): p. e53127.
119. Pope, H.G., Jr., et al., *Cognitive measures in long-term cannabis users.* J Clin Pharmacol, 2002. **42**(11 Suppl): p. 41S-47S.
120. Zvolensky, M.J., et al., *Prospective associations between cannabis use, abuse, and dependence and panic attacks and disorder.* J Psychiatr Res, 2008. **42**(12): p. 1017-23.
121. Zvolensky, M.J., et al., *Marijuana use and panic psychopathology among a representative sample of adults.* Exp Clin Psychopharmacol, 2010. **18**(2): p. 129-34.
122. van Ours, J.C., et al., *Cannabis use and suicidal ideation.* J Health Econ, 2013. **32**(3): p. 524-37.
123. Sewell, R.A., J. Poling, and M. Sofuoglu, *The effect of cannabis compared with alcohol on driving.* Am J Addict, 2009. **18**(3): p. 185-93.
124. Robbe, H.W.J. and O.H. J.F., *Marijuana and Actual Driving Performance. National Highway Traffic Safety Administration* 1993, U. S. Department of Transportation, DOT HS 808 078 November, 1993.
125. MacDonald, S., et al., *Driving behavior under the influence of cannabis or cocaine.* Traffic Inj Prev, 2008. **9**(3): p. 190-4.
126. Asbridge, M., J.A. Hayden, and J.L. Cartwright, *Acute cannabis consumption and motor vehicle collision risk: systematic review of observational studies and meta-analysis.* BMJ, 2012. **344**: p. e536.
127. Elvik, R., *Risk of road accident associated with the use of drugs: A systematic review and meta-analysis of evidence from epidemiological studies.* Accid Anal Prev, 2012.
128. Simpson, H.M., *Epidemiology of road accidents involving marijuana. .* Alcohol Drugs and Driving., 1986. **2**: p. 15-30.
129. Simpson, H.M., D.R. Mayhew, and R.A. Warren, *Epidemiology of road accidents involving young adults: alcohol, drugs and other factors.* Drug Alcohol Depend, 1982. **10**(1): p. 35-63.
130. Grotenhermen, F., et al., *Developing limits for driving under cannabis.* Addiction, 2007. **102**(12): p. 1910-7.
131. Fischer, B., et al., *Lower Risk Cannabis use Guidelines for Canada (LRCUG): a narrative review of evidence and recommendations.* Can J Public Health, 2011. **102**(5): p. 324-7.
132. Substance Abuse and Mental Health Services Administration, *Results from the 2013 National Survey on Drug Use and Health: Summary of National Findings , NSDUH Series H-48*. 2014, Substance Abuse and Mental Health Services Administration,: Rockville, MD.

133. Anthony, J.C., L.A. Warner, and R.C. Kessler, . *Comparative epidemiology of dependence on tobacco, alcohol, controlled substances, and inhalents: basic findings*
from the National Comorbidity Survey. E. Exp Clin Psychopharmacol, 1994. **2**: p. 244-268.
134. Jones, R.T., *Cardiovascular system effects of marijuana.* J Clin Pharmacol, 2002. **42**(11 Suppl): p. 58S-63S.
135. Arora, S., et al., *ST-segment elevation myocardial infarction in a 37-year-old man with normal coronaries--it is not always cocaine!* Am J Emerg Med, 2012. **30**(9): p. 2091 e3-5.
136. Charles, R., S. Holt, and N. Kirkham, *Myocardial infarction and marijuana.* Clin Toxicol, 1979. **14**(4): p. 433-8.
137. Karabulut, A. and M. Cakmak, *ST segment elevation myocardial infarction due to slow coronary flow occurring after cannabis consumption.* Kardiol Pol, 2010. **68**(11): p. 1266-8.
138. Singh, N.N., et al., *Cannabis-related stroke: case series and review of literature.* J Stroke Cerebrovasc Dis, 2012. **21**(7): p. 555-60.
139. Wolff, V., et al., *Cannabis-related stroke: myth or reality?* Stroke, 2013. **44**(2): p. 558-63.
140. Bloom, J.W., et al., *Respiratory effects of non-tobacco cigarettes.* Br Med J (Clin Res Ed), 1987. **295**(6612): p. 1516-8.
141. Tashkin, D.P., et al., *Effects of habitual use of marijuana and/or cocaine on the lung.* NIDA Res Monogr, 1990. **99**: p. 63-87.
142. Baldwin, G.C., et al., *Marijuana and cocaine impair alveolar macrophage function and cytokine production.* Am J Respir Crit Care Med, 1997. **156**(5): p. 1606-13.
143. Tashkin, D.P., et al., *Heavy habitual marijuana smoking does not cause an accelerated decline in FEV1 with age.* Am J Respir Crit Care Med, 1997. **155**(1): p. 141-8.
144. Taylor, D.R., et al., *A longitudinal study of the effects of tobacco and cannabis exposure on lung function in young adults.* Addiction, 2002. **97**(8): p. 1055-61.
145. Tashkin, D.P., *Smoked marijuana as a cause of lung injury.* Monaldi Arch Chest Dis, 2005. **63**(2): p. 93-100.
146. Hashibe, M., D.E. Ford, and Z.F. Zhang, *Marijuana smoking and head and neck cancer.* J Clin Pharmacol, 2002. **42**(11 Suppl): p. 103S-107S.
147. Hashibe, M., et al., *Epidemiologic review of marijuana use and cancer risk.* Alcohol, 2005. **35**(3): p. 265-75.
148. Allen, J.H., et al., *Cannabinoid hyperemesis: cyclical hyperemesis in association with chronic cannabis abuse.* Gut, 2004. **53**(11): p. 1566-70.
149. Sullivan, S., *Cannabinoid hyperemesis.* Can J Gastroenterol, 2010. **24**(5): p. 284-5.
150. Gessford, A.K., et al., *Marijuana induced hyperemesis: a case report.* W V Med J, 2012. **108**(6): p. 20-2.
151. Klein, T.W. and C.A. Newton, *Therapeutic potential of cannabinoid-based drugs.* Adv Exp Med Biol, 2007. **601**: p. 395-413.

152. Hosking, R.D. and J.P. Zajicek, *Therapeutic potential of cannabis in pain medicine.* Br J Anaesth, 2008. **101**(1): p. 59-68.
153. Abrams, D.I., et al., *Short-term effects of cannabinoids in patients with HIV-1 infection: a randomized, placebo-controlled clinical trial.* Ann Intern Med, 2003. **139**(4): p. 258-66.
154. Abrams, D.I., et al., *Cannabis in painful HIV-associated sensory neuropathy: a randomized placebo-controlled trial.* Neurology, 2007. **68**(7): p. 515-21.
155. Nong, L., et al., *Altered cannabinoid receptor mRNA expression in peripheral blood mononuclear cells from marijuana smokers.* J Neuroimmunol, 2002. **127**(1-2): p. 169-76.
156. Yuan, M., et al., *Delta 9-Tetrahydrocannabinol regulates Th1/Th2 cytokine balance in activated human T cells.* J Neuroimmunol, 2002. **133**(1-2): p. 124-31.
157. Bredt, B.M., et al., *Short-term effects of cannabinoids on immune phenotype and function in HIV-1-infected patients.* J Clin Pharmacol, 2002. **42**(11 Suppl): p. 82S-89S.
158. Hegde, M., et al., *Seizure exacerbation in two patients with focal epilepsy following marijuana cessation.* Epilepsy Behav, 2012. **25**(4): p. 563-6.
159. Fatma, H., et al., *Cannabis: a rare cause of acute pancreatitis.* Clin Res Hepatol Gastroenterol, 2013. **37**(1): p. e24-5.
160. Howaizi, M., et al., *Cannabis-induced recurrent acute pancreatitis.* Acta Gastroenterol Belg, 2012. **75**(4): p. 446-7.
161. Rawal, S.Y., D.N. Tatakis, and D.A. Tipton, *Periodontal and oral manifestations of marijuana use.* J Tenn Dent Assoc, 2012. **92**(2): p. 26-31; quiz 31-2.
162. Day, N.L., et al., *Effect of prenatal marijuana exposure on the cognitive development of offspring at age three.* Neurotoxicol Teratol, 1994. **16**(2): p. 169-75.
163. Fried, P.A., *Prenatal exposure to tobacco and marijuana: effects during pregnancy, infancy, and early childhood.* Clin Obstet Gynecol, 1993. **36**(2): p. 319-37.
164. Goldschmidt, L., N.L. Day, and G.A. Richardson, *Effects of prenatal marijuana exposure on child behavior problems at age 10.* Neurotoxicol Teratol, 2000. **22**(3): p. 325-36.
165. Kozer, E. and G. Koren, *Effects of prenatal exposure to marijuana.* Can Fam Physician, 2001. **47**: p. 263-4.
166. Hathaway, A.D., N. Comeau, and P.G. Erickson, *Cannabis normalization and stigma: Contemporary practices of moral regulation.* Criminology and Criminal Justice, 2011. **11**(5): p. 451–469.
167. Palamar, J.J., M.V. Kiang, and P.N. Halkitis, *Development and psychometric evaluation of scales that assess stigma associated with illicit drug users.* Subst Use Misuse, 2011. **46**(12): p. 1457-67.
168. Health Canada, *Information for Professionals: (Cannabis, marihuana) and the cannabinoids*. 2013: Ottawa.
169. Zuurman, L., et al., *Pharmacodynamic and pharmacokinetic effects of the intravenously administered CB1 receptor agonist Org 28611 in healthy male volunteers.* J Psychopharmacol, 2009. **23**(6): p. 633-44.

170. Watts, B.V., et al., *Meta-analysis of the efficacy of treatments for posttraumatic stress disorder*. J Clin Psychiatry, 2013. **74**(6): p. e541-50.
