# Supplementary material for: The short-term impact of 3 smoked cannabis preparations versus placebo on PTSD symptoms: A randomized cross-over clinical trial
Source: PLoS One. 2021 Mar 17;16(3):e0246990. doi: 10.1371/journal.pone.0246990 (PMC7968689; doi:10.1371/journal.pone.0246990)
Supplement: Attachment — Submitted filename: Response to Reviewers.docx [file pone.0246990.s016.docx]

RESPONSE TO REVIEWER FEEDBACK

Thank you for including your competing interests statement. Please confirm that this does not alter your adherence to all PLOS ONE policies on sharing data and materials, by including the following statement: "This does not alter our adherence to  PLOS ONE policies on sharing data and materials.” (as detailed online in our guide for authors http://journals.plos.org/plosone/s/competing-interests).  If there are restrictions on sharing of data and/or materials, please state these. Please note that we cannot proceed with consideration of your article until this information has been declared.

*We have added the statement “This does not alter our adherence to PLOS ONE policies on sharing data and materials” to the disclosures.*

Please include your updated Competing Interests statement in your cover letter; we will change the online submission form on your behalf.

*Thank you!*


**Comments to the Author**

Reviewer #1: This study examined the efficacy of 3 formulations of smoked cannabis versus placebo on PTSD symptoms over a three week period. There was no difference between active conditions and placebo on the primary outcome, and all conditions including placebo showed significant improvements in PTSD over a three week period.

This project is justified as there is a serious lack of RCTs on the effects of cannabinoids in PTSD, although pre-clinical and anecdotal evidence are numerous.

Report includes the protocol link, ethics approval, written consent information, detailed statistical analysis section and power analysis.

Major comments-

The findings that there is a high placebo effect is explained by the authors as being due to high expectations or bias by participants on cannabis's efficacy, and by the fact that the placebo blind was upheld. As the authors mention, the use of a 'Beliefs / attitudes toward efficacy of cannabis' measure would have been useful to control for this.

The authors also acknowledge that the lesser quality of cannabis used for the study was likely responsible for the possibly sub-therapeutic amounts consumed. This is a concern for the overall usefulness of the research, given that some participants may have consumed too little of the compounds to elicit any effect. The use of an ad libitum administration approach is potential flaw in this study, although setting a minimum volume to consume may also be problematic.

*We completely understand this reviewer’s concerns, as we initially weighed the pros and cons of ad libitum versus standardized dosing as well. Ultimately, the choice of using ad libitum dosing was based on previous literature showing large between subject variability in dose-response for cannabinoids, and data suggesting that participants will self-titrate based on tolerability. In this study, participants were instructed to smoke “ad libitum” with an upper maximum of 1.8 grams/day. This upper limit was necessary due to the outpatient setting for self-administration and the Schedule 1 controlled substance status of the investigational product. In reading this reviewer’s concerns we realized that we never provided our justification for this choice, and have added the following to the introduction:*

*“In addition, previous studies rely entirely on standardized dosing, rather than using more naturalistic and generalizable ad libitum dosing regimens. This is a major limitation of previous research because there is substantial individual variability in cannabinoid tolerability[29]. Indeed, military veterans who use cannabis for PTSD tend to self-titrate to much larger doses than those tested in previous trials[30,31].”*

Given that the participants may have consumed too little of the IP, with possible high expectations of the efficacy of the IP, and all groups showing a similar improvement on PTSD symptoms after a short time, this makes it difficult to draw any meaningful conclusions from the data. These limitations are openly acknowledged and well-discussed, and future studies are clearly necessary to overcome these limitations.

*We agree with this reviewer’s concerns. This was the very first randomized, blinded clinical trial to ever test whole plant cannabis as a treatment for PTSD. The overarching goal was to collect data on safety, determine whether there was a preliminary signal for efficacy in one or all cannabinoid formulations, and collect data on what modifications might be needed in future trials that test whole plant cannabis with appropriate statistical power. While our data cannot definitely tell readers whether cannabis is helpful or harmful for PTSD, we believe that our findings provide the information necessary for designing future trials and are consistent with the goals of an early stage RCT.*

Minor Concerns-

Consider placement of some citations in introduction (after full stops/ commas)

*We have updated the references so that they are consistent with the journal style.*

Statistics could be better presented, particularly the secondary outcomes.

*To make the results clearer, we only included information in the text of the results section on whether the omnibus between groups tests were significant for all secondary analyses. This helped to reduce redundancy within the results section, as this data is already summarized in the tables, and emphasized to the reader what outcomes were and were not significant between groups.*

Reviewer #2: This study, although in many respects well designed and performed, suffers from difficulties in interpreting the results due complicating factors and the lack of effective blinding. As the authors discuss (although this should have been made clear in the Methods section), the study recruited exclusively active cannabis users, therefore time-varying withdrawal symptoms in the subsequent abstention periods and during the intervention are likely to play a role. Although the study is described as a cross-over, this is not a balanced cross-over design and placebo is lacking in the second stage, therefore the usual analysis methods for crossover studies are not used. Despite a wash-out period, analysing the second stage is complicated by possible carryover effects from the first-stage intervention. The second-stage results are in any case rather incompatible with those of the first stage. The lack of successful blinding in a trial with subjective outcomes (either participant or clinician reported) means that any observed before-after or group comparison effects cannot be unambiguously assigned to real effects of the interventions.

For future trials it would be important to consider whether objective outcomes are available or, failing this, whether the outcome assessors could be more effectively and convincingly blinded.

*We are very grateful for this comprehensive feedback. To address the reviewer’s concerns that were not discussed in our Discussion section as limitation, we made the following modifications::*

1) *Included information on recruitment methods in the Methods section under Participants (Page 5).*
2) *Included suggestions on ways to mitigate the blinding issues in the Discussion section (Page 32)*

Minor

1. Describe recruitment methods in the methods section

*We have included the following information under Participants (Page 5):*

*The study recruited male and female US military veterans with PTSD through community-based advertisements, presentations, and website advertisements.*

2. Why were there unequal assignments (n=29/27/18) to arms in stage 2: each arm re-randomised independently?

*This was due to a limitation of the study design. Per protocol, participants were randomized 1:1:1:1 in Stage 1 across four groups. After Stage 1, participants abstained from using product for 2 weeks during Cessation 1, and then they were re-randomized in a blinded manner into 2 of 3 active marijuana dose groups with a 1:1 ratio, while excluding their prior randomized condition in Stage 1. As placebo was not an option in Stage 2, placebo participants were randomized 1:1 between High THC and High CBD, but were not given the option to be randomized to THC + CBD in order to facilitate simpler programming of the web-based randomization system. This two-step randomization resulted in an unbalanced distribution of Stage 2 participants overall across groups, but each individual group had a balanced 1:1 likelihood randomization scheme in Stage 2. Due to anticipated dropouts in the Cessation period following Stage 1, the protocol states: “Due to enrollment and dropouts to achieve N=76 for primary endpoint, the actual number of Stage 2 participants may be higher or lower.” We have included a description of this under Randomization and Blinding methods and updated the Consort Diagram (Figure 1) to show how participants were re-randomized in Stage 2, as well as the accrued subjects and dropouts by each study period. A description of this limitation has also been added to the discussion.*

3. What is the p-value (left side) in Table 2?

*This first column was for the total sample, but given that only the baseline differences between groups are relevant for testing we have deleted the first column of p-values to make this clearer.*

4. Treatment preferences are described on p.14, but we need to know which choice of interventions was offered (e.g. so-and-so many preferred High THC to THC+CBD, etc.)

*We agree that it would informative to know treatment preferences for each combination of drug. However, we chose not to report the individual treatment preferences of each combination because there were 8 full treatment combinations, with three potential categories of preference for each combination (stage 1 drug, stage 2 drug, or equal preference), meaning that there would be 24 cells for just 74 participants. Therefore, we chose instead to report the percentage of participants who preferred each treatment if it was one of the treatments that they received.*

5. I suggest including a table showing the numbers crossing over from each stage 1 intervention to each stage 2 Intervention

*We agree that having a visual representation of this would be helpful and have added this information to Figure 1.*

6. Tab. 3 needs a legend: state that the numbers in the table refer to numbers of participants, not events.

*We have added a notes/legend section at the end of the table that states that the counts/frequencies represent the number of participants reporting that AE.*

7. Discussion p.30: why is the first reason given (low cannabis exposure) an explanation for the observed placebo effect?

*Upon re-reading, we agree that the ordering of this section doesn’t make sense. We have changed the first paragraph of this section (pg. 30) to now read:*

*“The study’s failure to detect a significant difference between groups in Stage 1 could perhaps be explained by several confounding factors. First, the study sample included participants with a history of cannabis use. The recruitment of active cannabis users might have increased the potential for biased responding. Given the topical nature of the current trial and its relevance for public policy on medical cannabis, participants might have been biased to report positive effects regardless of condition. Despite many participants already having experience with the drug, nearly half of those receiving placebo believed that they received active cannabis. Prior expectations about cannabis’ effects might explain why even those in the placebo condition reported larger than average reductions in PTSD symptoms after only 3 weeks of treatment.*

8. Discussion p.31: interpretation of stage 2 results is unreliable due to possible carry-over effects and to the inconsistencies compared to stage 1.

*We have removed the more thorough discussion of patterns of observed effects in Stage 2 from pg 31, and instead added additional information on safety outcomes, as safety analysis was a primary aim of the study. We also added a sentence to the discussion that all stage 2 results should be interpreted with caution.*

9. Fig 1: 80, not 261, were randomized

*We have updated Figure 1 to correctly list that 80 participants were randomized in Stage 1.*

10. Fig 2 & 3. error bars unclear due to overlap – I suggest staggering them by slightly displacing them on the x-axis.

*We have updated Figures 2 & 3 so that the error bars between groups do not overlap.*

11. Warning: supplementary included datasets are not anonymous: initials and date of birth are included.

*We are very grateful to the reviewer for catching this! All supplementary datasets are now appropriately de-identified.*
