# Supplementary material for: The short-term impact of 3 smoked cannabis preparations versus placebo on PTSD symptoms: A randomized cross-over clinical trial
Source: PLoS One. 2021 Mar 17;16(3):e0246990. doi: 10.1371/journal.pone.0246990 (PMC7968689; doi:10.1371/journal.pone.0246990)
Supplement: Attachment — Submitted filename: Responses to Feedback_Revision2.docx [file pone.0246990.s017.docx]

RESPONSE TO REVIEW

Reviewer #3: This is my first review of this manuscript (now in revised form) describing the outcomes of a double-blind randomized placebo control trial with two stages and three varieties of inhaled whole plant cannabis on PTSD symptoms in a sample of military veterans.

Why was there no placebo control in Stage 2?

*This was a question that was also raised by previous reviewers and we might not have fully explained the process of how we determined our design. Stage 1 was designed to test our primary efficacy aim and Stage 2 was intended to be entirely exploratory. Unfortunately, we didn’t anticipate that placebo would have a pronounced effect, so we assumed that we would not need a full cross-over analysis to test for differences from placebo.* *There were also concerns about retention in a clinical drug trial with a population suffering from severe psychiatric symptoms when the intervention wasn’t standard of care. We chose the end of Stage 1 as the primary endpoint of the study to minimize the potential for missing data, assuming that we would see significant drop out between the Stages. The rationale for including a Stage 2 was to allow participants to compare their experience on the two preparations they received in the study to gauge participant preference between the two Stages.*

Please avoid the use of noun labels (PTSD patients) and replace with the less stigmatizing person first labels (patients with PTSD).

*Thank you for this suggestion. We have changed this throughout the manuscript.*

A recent paper has examined the therapeutic effects of inhaled cannabis flower and concentrates on PTSD symptoms using a naturalistic design with ad libitum dosing (see LaFrance et al., 2020; JAD). This recent paper should be acknowledged or the statement that “the potential therapeutic effects of cannabinoids on PTSD have not been examined with smoked, herbal cannabis” should be modified. The statement regarding the failure of previous studies to examine ad libitum dosing could also be slightly modified to reflect this recent contribution to the literature.

*We thank the reviewer for suggesting the inclusion of this reference. We have added information on the suggested paper into the background and discussion sections of the article and* *changed the following sentence to now read “…the potential therapeutic effects of smoked, herbal cannabis on PTSD have not been examined in a randomized, placebo controlled trial.”*

If “military veterans who use cannabis for PTSD tend to self-titrate at much larger doses” then the required use of an upper limit may be diminishing the power of the study to detect significant effects. I understand this was required but it should be discussed as a limitation. The use of relatively low potency cannabis could also be offered as a limitation (albeit a necessary one given the limited products produced by the NIDA drug supply). I am particularly intrigued about the finding that the 12% THC product supplied by NIDA tested at a mere 9%!

*We noted in the discussion section that the NIDA limit might have created an artificial ceiling. However, it is worth noting that while an upper limit was imposed by regulatory bodies, the majority of participants in the study did not use the full amount of cannabis given to them.*

The sample is predominantly male, and this should be identified as a limitation. Especially in light of recent research (Lafrance et al., 2020) indicating that females may report larger reductions in PTSD symptoms than males after cannabis use and that females are more likely to experience PTSD than males.

*This is an excellent suggestion. Owing to the population that we were sampling from (military veterans), the sample was predominantly male. We have added this to the limitations section for generalizability to PTSD broadly.*

Please describe the nature of the 3 SAE reported during Stage 2. Also please define SAE for the reader upon its first instance.

*We have added the definition of serious adverse event (SAE) in the first instance. We re-ordered some of the information in the AE/SAE summary paragraph because the SAEs actually occurred across both stages. We have added the information on what each SAE was and when in the study it occurred and also updated the Consort figure to make clear where drops/withdraws occurred throughout the trial, as many occurred during the washout periods.*

It would seem more appropriate to use mixed factorial ANOVA with symptom severity as the DV, time (baseline, end of treatment) as a within-subjects factor and treatment condition (THC, CBD, THC+CBD, placebo) as a between-subjects factor (rather than relying on change scores for the DV). This might better help to control for/consider variability in symptom severity at baseline (and potentially pull out some trends the authors are detecting). The authors can then report the main effect of time, treatment, and the interaction between these variables. Relatedly, I may be missing something, but it is not clear why MMRM was used for the subscale analyses but not the primary data analyses.

*These are excellent suggestions. Consistent with guidance from FDA, one of the ways that we’re attempting to reduce type 1 error is to adhere to our statistical analysis plan that was finalized before study start. This unfortunately means that we are limited in our ability to adjust our analytic plan for the primary outcome posthoc.*

*Regarding the use of MMRM for only a subset of variables, the CAPS was only administered at two timepoints, whereas the PCL, ISI, and IDAS were administered at multiple timepoints. Multiple administration with the PCL, ISI, and IDAS allowed us to use a more robust approach.*

Ideally the direction of treatment effects in Stage 2 would be reported in text as well. This is done nicely for the PCL-5 section but not in the CAPS-5 or Post hoc results sections. While the reader can determine this for themselves using the tables that requires considerably more time and cognitive resources on the part of the reader.

*In the last round of review our reviewers suggested that we reduce redundancy by keeping results either in text or in the tables. To best accomplish this, we elected to move the majority of Stage 2 results only to the Tables because Stage 2 was an exploratory stage. We do agree that a brief summary of results for each endpoint would be helpful, though, particularly in explaining any significant results. To balance the feedback from the reviewers, we have added back some of the additional information on Stage 2 results, which summarize the simple main effects that explain all significant omnibus findings.*

I suggest you cite research showing that chronic cannabis users can test positive for the drug after even a month of abstinence in paragraph 3 of the discussion. Also, in the same paragraph the authors suggest that cessation of cannabis use in the placebo group could have produced the change in symptoms, but this interpretation doesn’t make a lot of sense since scores were decreased in this group and in the cannabis groups. Why would both removing and continued use of a drug produce the same effect?

*We have added a sentence to paragraph 3 stating that cannabis metabolites can remain in urine for up to 30+ days after cessation of use.*

*The reviewer asks an excellent question about one potential limitation that we raised in the manuscript for readers to consider. Since all groups reported at least moderate cannabis withdrawal symptoms at baseline, those randomized to placebo would have experienced continued abatement of their withdrawal symptoms throughout phase 1. This could have been subjectively experienced as alleviation or reduction in distress, which overlaps with psychological symptom measures. Likewise, participants randomized to active THC could have also experienced a reduction in withdrawal through drug exposure. In both scenarios, the previous withdrawal symptoms could potentially confound or overlap with reported reductions in PTSD symptoms.*

Participants may have found the NIDA drug “harsher” because NIDA literally uses the whole plant rather than the buds sold in dispensaries. The differences in these products could be further detailed in the discussion.

*We appreciate this comment! We have added a more detailed discussion of the potential issues with using the NIDA cannabis supply in clinical trials.*

Minor Issues

There is a typo with the parentheses in the abstract. In the abstract (and Interventions section in the body of the paper) the information about the cannabinoid content of the placebo should be in separate parentheses after “compared to placebo” rather than in the parentheses describing the three active concentrations.

*We appreciate the catch. This has been fixed.*

The abstract should contain the sample sizes for each stage.
*We have added the sample sizes for each stage to the abstract.*

It would be best to define the acronym DoD/VA
*We have added the description for Department of Defense (DoD) and Veterans Affairs (VA)*

The link to the trial protocol does not work.

*We have updated the link: https://maps.org/research-archive/mmj/MJP1-Protocol-Amend4-oct-13-2015.pdf*

There are grammatical issues with these two sentences “The study recruited male and female US military veterans with chronic PTSD through community-based advertisements, presentations, and website advertisements. The study include participants based on the following inclusion and exclusion criteria”

*These sentences now read “Study participants were recruited using community-based advertisements, presentations, and website advertisements. Study inclusion and exclusion criteria were as follows:”*

The requirement of being a military veteran appears twice in the inclusion criteria (1 & 4)
*This has been corrected.*

It should be stated whether or not emergency unblinding was ever required.
*Emergency unblinding was never required. This has been added to the paragraph on AE descriptions.*

The abbreviations used in the first column of Table 1 should be defined under the table.
*We have added the abbreviations with their definitions to the key at the bottom of Table 1.*

“Enrollment and randomization continued until 76 participants completed the Stage 1
outcome assessment (N = 80).” Why is N = 80 and not N = 76 in the parentheses? This confusion appears later in the manuscript as well (Statistical analyses section). I think these issues could be resolved by moving the Sample Characteristics section up to appear after (or within) the Participants section.

*We appreciate that the reviewer noticed this and identified and fixed the inconsistencies of reporting N throughout the paper. Specifically, we changed the N to 76 in the parengtheses after the sentence that the reviewer noted because only 76 participants completed outcome assessments at the end of Stage 1. We also added columns to all results tables for the N associated with the completed data that were analyzed in the models. We believe that this will make it clearer for the reader to interpret how many participants completed baseline assessments and whether this N was different than the number of assessments that were analyzed.*

The type of effect size used (eta squared, Cohen’s f), the power value used (presumably .80) and alpha level considered should be included in the description of the power analysis.

We have added the following information to the study power section on page 10:

*Power analysis suggested that 76 completing participants (n = 19 per group) would be needed to detect an effect size of d = 0.4 at 82% power and .05 significance level. Enrollment and randomization continued until 76 participants completed the Stage 1 outcome assessment. Eighty participants were enrolled and 76 partcipants completed Stage 1.*

Given the length of Table 3 the headers should be repeated on each new page.

*Headers have been added to the top of each page of Table 3.*
